# Restoring Histone Acetylation Accelerates Diabetic Wound Repair by Improving the Spatiotemporal Dynamics of Macrophages

**DOI:** 10.1002/advs.202504920

**Published:** 2025-10-06

**Authors:** Karmveer Singh, Albert Kallon Koroma, Rajeev Kumar Pandey, Yongfang Wang, Jinnan Cheng, Philipp Haas, Mahyar Aghapour, Linda Krug, Adelheid Hainzl, Meinhard Wlaschek, Pallab Maity, Karin Scharffetter‐Kochanek

**Affiliations:** ^1^ Department of Dermatology and Allergic Diseases Ulm University 89081 Ulm Germany; ^2^ Aging Research Center (ARC) 89081 Ulm Germany; ^3^ Bloomberg∼Kimmel Institute for Cancer Immunotherapy, Sidney Kimmel Comprehensive, Cancer Center, Department of Oncology Johns Hopkins University School of Medicine Baltimore MD 21287 USA

**Keywords:** chronic wounds, diabetes, dysfunctional macrophages, epigenetic regulation, persistent inflammation

## Abstract

Dysregulation of macrophage populations at the wound site is responsible for the non‐healing state of chronic wounds. The underlying mechanisms in diabetic conditions at single cell resolution and therapeutic advances remain, however, largely unexplored. Here, it is reported that acetyl histone‐H3 (Lys27), an epigenetic mark regulating the macrophage transcriptome, is lost in the hostile tissue microenvironment of diabetes. Diabetic conditions suppress the acetylation of histone, a critical regulator of trained immunity, by activating histone deacetylase (HDAC)‐dependent deacetylation pathways. This, in consequence, suppresses STAT1 signaling in macrophages under diabetic conditions. Interestingly, butyrate, a potent HDAC inhibitor, restores the acetyl histone‐H3 (Lys27)‐dependent transcriptome and thereby effectively rescues macrophage functions even in a persisting diabetic microenvironment. Butyrate not only reinstalls the physiologic STAT1 mediated immune program in macrophages at early phases of diabetic skin repair, but also harmonizes macrophage interactions with keratinocytes and fibroblasts, depicting a unique fingerprint of tissue regeneration. Most interestingly, butyrate breaks the vicious cycle of inflammation in chronic wounds by restoring classical stages of wound healing. This study offers novel pathogenic insight and the unique opportunity to reverse perturbed immune function, holding promise to successfully treat diabetic and other chronic wounds with unresolved inflammation.

## Graphical abstract

Butyrate—via epigenetic modulation of the histone core—rebalance dysregulated macrophage function and promotes wound healing under persisting hostile conditions of diabetic mice. Additionally, butyrate harmonizes macrophage interactions with keratinocytes and fibroblasts as well as breaks the vicious cycle of inflammation in chronic wounds by restoring classical kinetics of wound healing.

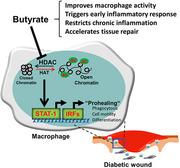

## 1 Introduction

Metabolic disorders like obesity and diabetes are often associated with life threatening secondary complications such as perturbed immune responses, angiopathy, and impaired wound healing.^[^ ^1^, ^2^, ^3^, ^4^ ^]^ Obesity and diabetes enforce profound changes in the quantity and quality of the transcriptome of most cell types, including distinct immune cell populations.^[^ ^5^, ^6^ ^]^

Tissue repair is vital for organ integrity and proceeds through a highly coordinated and overlapping phases like inflammation, proliferation, and remodeling, depending on complex interactions between a variety of immune and non‐immune cells.^[^ ^7^ ^]^ However, chronic wounds fail to progress through the physiologic pattern of wound repair, but instead remain in an ineffective, persisting inflammatory state with little signs of healing.^[^ ^8^, ^9^, ^10^, ^11^, ^12^ ^]^ Perturbed macrophage function represents a hallmark of non‐healing diabetic wounds^[^ ^13^ ^]^ and other chronic wounds.^[^ ^14^, ^15^ ^]^ Despite robust expansion of macrophages in obese adipose tissue,^[^ ^5^, ^6^, ^16^ ^]^ this and other macrophage populations fail to be recruited and to be activated in a highly controlled manner at the wound site, leading to unresolved inflammation in non‐healing wounds. Recent studies indicate that macrophages likely impact on other cells at the wound site and even support keratinocyte migration and the formation of new blood vessels.^[^ ^10^, ^17^ ^]^

Increasing evidence demonstrates the importance of epigenetics in macrophage regulation and its disruption—due to metabolic disorders—may even be responsible for several pathologies and impaired tissue repair.^[^ ^15^, ^18^, ^19^, ^20^ ^]^ Therefore, studies on epigenetics in metabolic disorders have attracted increasing attention,^[^ ^21^ ^]^ and evidence was forwarded in general that metabolites may influence the transcriptional program of genes by affecting methylation, acetylation, and phosphorylation of chromatin.^[^ ^22^ ^]^

The epigenetic machinery controls gene activity through post‐translational modifications, including acetylation, methylation, phosphorylation, and ubiquitylation, either on DNA or tails of histone proteins.^[^ ^23^, ^24^ ^]^ Increasing evidence demonstrates the importance of epigenetics in macrophage regulation and—if disrupted due to metabolic disorders—may even be responsible for several pathologies and impaired tissue repair.^[^ ^15^, ^18^, ^19^, ^20^ ^]^ Both beneficial and detrimental effects of histone modifications on macrophage function and polarity have been described.^[^ ^25^, ^26^, ^27^, ^28^ ^]^ Recent reports even suggest a critical role of epigenetic marks in macrophage polarization, maintaining memory against pathogen as earlier only known for adaptive immunity.^[^ ^29^, ^30^ ^]^

So far, neither the underlying mechanisms of metabolic dysregulation impacting on the transcriptomic landscape and macrophage heterogeneity, nor its potential rescue through epigenetic restoration towards normal genomic signatures and appropriate macrophage functions have been addressed in sufficient detail in diabetic wounds. It would be of high clinical relevance to explore whether the restoration of macrophage epigenetics would foster healing of chronic diabetic wounds. Here we show that the HDAC inhibitor, butyrate, accelerates diabetic wound healing by activating classical repair programs dominated by macrophages.

## 2 Results

### 2.1 Diabetes Impairs Tissue Homeostasis, Regeneration and the Immune Response

To identify diabetes associated changes in skin physiology and tissue repair, we have compared the whole transcriptome of non‐injured skin and full‐thickness skin wounds of the leptin receptor deficient *db/db* diabetes mouse model^[^ ^31^ ^]^ with age matched healthy wild‐type littermate control mice. For this purpose, RNA was isolated from intact or wounded skin from young male *db/db* and wild‐type mice, subsequently subjected to RNA‐seq and bioinformatic analysis.

**Figure 1:**
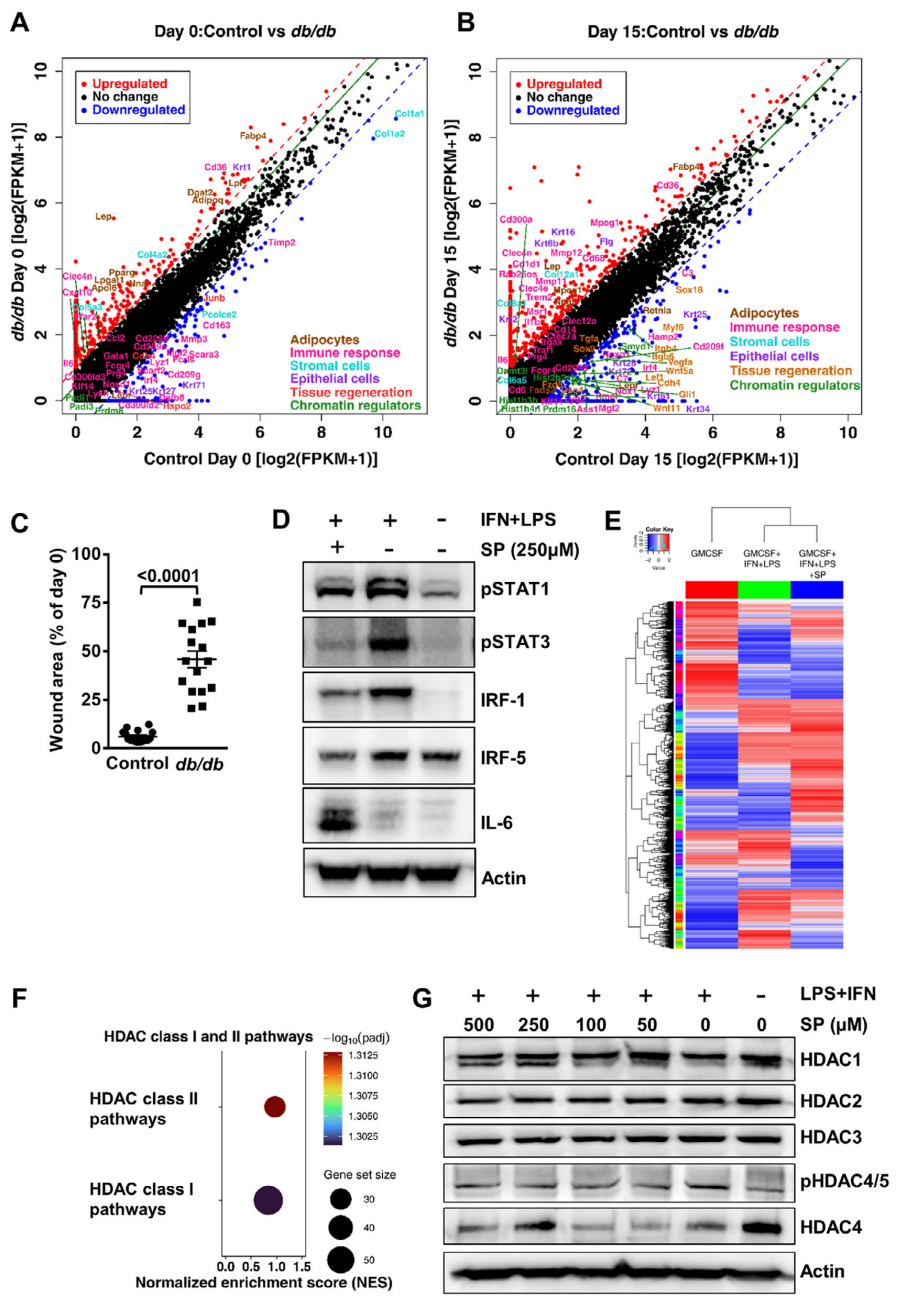
Diabetes globally remodels the transcriptomes of non‐injured skin and during repair. **A)** Scatter plot of gene expression fold change between uninjured wild type [x axis, log2 (FPKM+1) scale of wild type] and *db/db* mice skin [y axis, log2 (FPKM+1) scale of *db/db*], as well as between **B)** 15 days old skin wounds. Highly significant expression of genes is indicated by red (upregulated) and blue (downregulated) dots. p values were determined by the Mann‐Whitney test. **C)** Wound healing comparison between wild type (C57B6/J) control and *db/db* diabetic mice on day 15 post injury. Unpaired t‐test, values are represented as mean ± SEM, *n* = 4. **D)** Western blot analysis displaying expression of key proteins regulating inflammatory signaling after incubation of stimulated macrophages with 250 µm sodium palmitate (SP) for 18 h. These results are representative of three independent experiments. Human beta‐actin was used as a loading control. **E)** Heatmap demonstrating comparison of global transcriptomic profile between unstimulated, stimulated, and stimulated macrophages exposed to SP. Color key indicates fold change in gene expression. **F)** Dot plot showing upregulation of HDAC class I and class II pathways in stimulated macrophages following incubation with SP. **G)** Protein expression of HDAC family members in stimulated macrophages following exposure with increasing doses of SP. These results are representative of three independent experiments. Human beta‐actin was used as a loading control.

Analysis of gene signatures in non‐injured skin (D0) revealed a gain of gene expression of Fabp4, Dgat, Lpl, Adipoq, Pparg, Fabp4, Lgal1, Lep, Cd36, and Il6 (**Figure** **1**A), genes which are involved in lipogenesis. By contrast, a reduced transcriptome signature of genes like Col1α1, Col1α2, Padi1, Padi3, Prdm8, Gata1, Mgl2, JunB, Irf4, Prg4, Cd163, Cd300ld2, Cd209g, Krt71, Krt27, Fcgr4, Lepr, Mmp3, Timp3, and Rspo2 associated with lipid metabolism, immune response, stromal as well as epidermal maintenance and chromatin regulation was observed in diabetic mice skin (Figure 1A).

Newly restored wound tissue (D15) from diabetic mice demonstrates enhanced expression of Flg, Fabp4, Trem2, Clec4e, Msr1, Krt2, Krt16, Il1b, Il6, Cd68, Cd36, and Cd300a (Figure 1B) genes, which all contribute to immune remodelling. Of note, diabetic wounds displayed a profound suppression of a transcriptome signature physiologically linked to tissue regeneration, immune response and chromatin regulation including genes like Lef1, Wnt5a, Wnt11, Fzd5, Gli1, Sox9, Sox18, Itgb4, Itgb5, Cdh4, Krt25, Krt28, Krt34, Krt81, C3, C7, Mgl2, Ass1, Klf14, Prg4, Traf1, Lyz1, Irf4, Fcgr4, Nox1, Cd14, Cd6, Cd209f, Hist1h3b, Hist1h4n, Dnmt3l, Smyd1 and Prdm16 (Figure 1B). These data identify a global rewiring of gene expression and indicate that diabetic microenvironmental conditions not only affect skin physiology but dampen the classical regenerative signals and alter immune response. Together, these transcriptomic changes likely contribute to delayed wound healing in diabetic mice as compared to wild‐type mice (Figure 1C). Consistent with our results, two recent studies showed that macrophage subsets of the innate immune system undergo fundamental remodelling during obesity and diabetes.^[^ ^5^, ^6^ ^]^

The lack of insight how the innate immune system, and particularly macrophage remodelling, affects tissue repair in diabetes prompted us to investigate these cells in more detail. We therefore set out to analyse both stimulated and non‐stimulated human macrophages in the presence or absence of sodium palmitate (hereafter referred to as SP), a free saturated fatty acid that is increased in diabetes and closely mimics diabetic metabolism.^[^ ^32^, ^33^ ^]^ To explore whether enhanced SP concentrations directly impact on the transcriptome signature of inflammation and macrophage polarization, we subjected unstimulated (PBS) and stimulated (LPS, lipopolysaccharide and IFNγ, interferon‐gamma) macrophages to SP at a subtoxic concentration of 250 µm for 18 h (Figure S1A,B, Supporting Information). Granulocyte‐macrophage colony‐stimulating factor (GmCSF) was used to generate macrophages from blood monocytes. An increase in the mRNA expression of pro‐inflammatory macrophage markers, including TNFα and IL‐23 was observed, while anti‐inflammatory markers like IL‐10 and CD206 were suppressed upon incubation with SP (Figure S1C, Supporting Information). These findings indicate that SP influences the expression of genes linked to macrophage polarization.

Next, we analyzed the impact of SP concentrations on the expression of STAT family proteins, which are major regulators enforcing inflammatory activity of macrophages.^[^ ^34^ ^]^ Western blot analysis demonstrates substantial downregulation of pSTAT1 and pSTAT3 in SP as opposed to vehicle (PBS) treated stimulated macrophages (Figure 1D; Figure S2A, Supporting Information). Consistent with reduced pSTAT proteins, STAT downstream effectors such as interferon regulatory factor 1 and 5 (IRF‐1 and IRF‐5) were decreased following incubation of stimulated macrophages with SP (Figure 1D; Figure S2A, Supporting Information), confirming our findings from transcriptome analysis of murine skin from *db/db* mice. Interleukin‐6 (IL‐6), a major driver for secondary inflammation in obesity and diabetes,^[^ ^35^ ^]^ was increased in stimulated macrophages after SP exposure as opposed to vehicle‐treated or unstimulated macrophages (Figure 1D; Figure S2A, Supporting Information).

To uncover the molecular mechanisms underlying the deviation of SP‐induced signaling, comprehensive global transcriptome analysis of stimulated macrophages in the presence and absence of SP was performed. Bioinformatic analysis revealed profound changes in global gene transcription of SP‐treated macrophages as opposed to the untreated group (Figure 1E). Among others, we observed an upregulation of gene sets belonging to the HDAC class 1 and 2 (Figure 1F). We further confirmed these findings at the protein level by analyzing the expression of key HDACs and observed higher expression of HDAC1 and HDAC4, while HDAC2 and HDAC 3 remained unchanged (Figure 1G; Figure S2B, Supporting Information). The expression of pHDAC4/5 was reduced in SP‐treated macrophages as opposed to IFN+LPS group (Figure 1G; Figure S2B, Supporting Information), phosphorylation promotes cytoplasmic export thereby reducing their nuclear activity and conversely reduced phosphorylation indicates higher deacetylates activity. At 250 µm SP concentration, we observed optimal inhibition of STAT1 signaling, higher HDACs activity, and IL‐6 induction (Figure S3A,B, Supporting Information).

These data indicate that the molecular and epigenetic landscape essential for skin homeostasis, repair capacity and innate immune macrophage signaling is disrupted under diabetic conditions.

### 2.2 Butyrate‐Mediated Restoration of Histone Acetylation in Macrophages Accelerates Wound Healing in Murine Type 2 Diabetes

**Figure 2:**
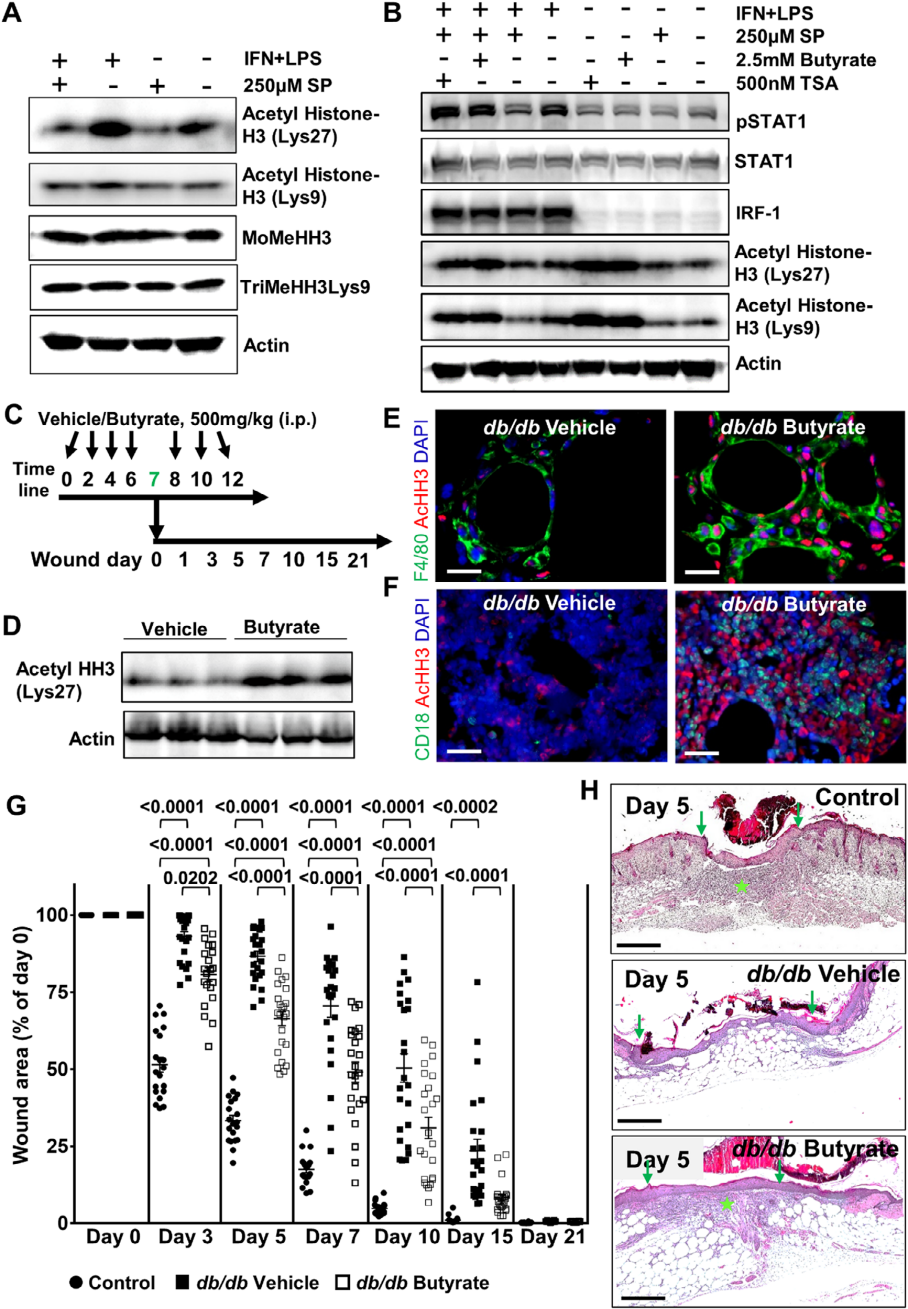
HDACs inhibition strongly restores macrophage signaling and accelerates wound healing in a hostile diabetic microenvironment. **A)** Expression of key acetylated and methylated histone modifications in resting or stimulated macrophages exposed to SP. Results are representative of three independent experiments. Human beta‐actin served as a loading control. **B)** Western blot analysis displaying the impact of HDAC inhibition by the pharmacological inhibitors butyrate and trichostatin A (TSA) on the STAT1 activation state (phosphorylation at Y701) in macrophages cultured under normal or SP. Enhanced expression of acetylated histone H3 is indicative of specific inhibition of HDAC by butyrate (2.5 mm) or TSA (500 nm). These results are representative of three independent experiments. Human beta‐actin was used as a loading control. **C)** The cartoon depicts the experimental design and administration schedule of the HDAC inhibitor butyrate or vehicle in *db/db* mice. **D)** Western blot shows marked increase in acetylated histone h3 (Lys 27) in skin lysates of *db/db* mice following butyrate administration. **E)** Representative immunostaining of 5 days old *db/db* mice wounds with macrophages marker F4/80 (green) and **F)** leukocytes stained with CD18 antibodies (green) within bone marrow, both demonstrating increased acetylated histone h3 (Lys 27) (red) following butyrate treatment as opposed to vehicle group (*n* = 5). Scale bars, 20 µm. **G)** Control and butyrate treated *db/db* mice display accelerated wound healing compared to vehicle treated *db/db* mice following 6 mm full‐thickness circular biopsy punch excision. One‐way ANOVA, values are represented as mean ± SEM, *n* = 5. **H)** Representative H&E photomicrographs of a 5‐day wound depicting enhanced (thickened) granulation tissue formation and marked matrix deposition (marked with star) in control and butyrate treated *db/db* mice compared to vehicle group. Scale bars, 500 µm.

We then investigated whether SP‐mediated activation of HDAC pathway interferes with the activity of epigenetic enzymes in macrophages. For this purpose, we analyzed the status of key histone modifications such as acetylation and methylation at histone tails. We identified a marked reduction of acetyl histone‐H3 (Lys27) in both unstimulated and stimulated macrophages following exposure to SP (**Figure** **2**A; Figure S4A, Supporting Information), supporting our findings at the transcriptional and protein level (Figure 1F,G). Acetyl histone‐H3 (Lys9) was also suppressed in macrophages, while the methylation marks remained unchanged after incubation with SP (Figure 2A; Figure S4A, Supporting Information).

To further validate our findings on whether suppressed acetyl histone‐H3 (Lys27) after SP exposure is causally linked to macrophage dysfunction, we specifically inhibited HDAC enzymes under SP conditions. We employed two different HDAC inhibitors, i.e., sodium butyrate (hereafter referred to as butyrate) and trichostatin A (TSA) to enhance the level of acetyl histone‐H3 (Lys27) in macrophages exposed to SP. Both butyrate and TSA‐mediated inhibition of HDAC rescued the pSTAT1 level in the presence of SP (Figure 2B; Figure S4B,C, Supporting Information). STAT1 activation following butyrate or TSA treatment even exceeded levels of STAT activation of stimulated macrophages (Figure 2B; Figure S4B,C, Supporting Information). Magnitude and specificity of butyrate treatment and SP exposure on HDAC were determined by the expression of acetyl histone‐H3 (Lys27 or Lys9) (Figure 2B; Figure S4B, Supporting Information). Both butyrate and TSA reversed the SP‐mediated suppression of histone‐H3 (Lys27 or Lys9) in stimulated macrophages (Figure 2B; Figure S4B, Supporting Information). The selected dose of the butyrate did not show any toxicity in the macrophages (Figure S4D, Supporting Information). Immunostaining against acetyl histone‐H3 (Lys27) depicts the restoration of nuclear staining of acetyl histone‐H3 (Lys27) in butyrate‐treated macrophages cultured under high SP conditions (Figure S5A, Supporting Information).

In a complementary Crispr epi‐editing approach, we performed locus (promoter)‐specific writing of acetyl histone‐H3 (Lys27) induced by dCas9‐histone acetyl transferase (HAT) (Figure S5B, Supporting Information). Enrichment of acetyl histone‐H3 (Lys27) targeting respective at IRF‐1 and S100‐A8 promoters rescued mRNA expression of these genes in macrophages exposed to SP (Figure S5B, Supporting Information).

These findings hint at an epigenetic regulation of STAT1 signaling in macrophages, which is lost under SP mimicking diabetic conditions and can effectively be restored to its original state by butyrate.

Next, we assessed whether our in vitro findings are of any clinical relevance for the in vivo situation of chronic diabetic wounds. Therefore, we repetitively administered 500 mg kg^−1^ butyrate intraperitoneally to the *db/db* mice starting before the induction of full‐thickness wounds, no toxicity has been reported in mice at this dose.^[^ ^36^, ^37^ ^]^ Butyrate treatment thereafter was continued during early wound healing stages (Figure 2C). We observed enhanced levels of acetyl histone‐H3 (Lys27) in skin lysates from butyrate treated *db/db* mice as opposed to vehicle‐treated group (Figure 2D). Continued treatment with butyrate also enhanced expression of histone‐H3 (Lys27) in wound macrophages (Figure 2E; Figure S5C, Supporting Information) as well as leukocytes lineages residing in bone marrow of diabetic mice (Figure 2F; Figure S5D, Supporting Information). Butyrate expanded the histone‐H3 (Lys27) positive CD18 (the common chain of β2 integrins), a pan leukocyte marker, expressed on the surface of all immune cell populations, including macrophages in the bone marrow as compared to the vehicle‐treated group (Figure 2F; Figure S5D, Supporting Information). These findings underscore an epigenetic regulation of macrophages and leukocytes.

Wound closure was significantly accelerated at days 3, 5, 7, 10, and 15 in mice that received butyrate as opposed to vehicle‐injected mice (Figure 2G). Thus, butyrate‐treated wounds revealed significantly enhanced healing kinetics. Vehicle treated age matched healthy control (C57B6/J) revealed significantly faster wound closure as opposed to both diabetic mice groups (Figure 2G). Faster wound closure in control skin is likely attributed by its regenerative stem cell pool, collagen‐rich dermis, higher contractile abilities, robust immune response, and lack of diabetes associated pathologies. Histologically, butyrate‐treated diabetic wounds and vehicle‐treated control wounds at day 5, indeed, displayed better matrix deposition and granulation tissue formation at the center of the wound site (marked with a star) as compared to vehicle‐treated diabetic wounds, where granulation tissue formation was limited to wound edges (Figure 2H). These findings indicate that restoring the acetylation state of histone core through repetitive butyrate administration expedites the diabetic wound healing process, enforcing deposition of extracellular matrix proteins at the wound site, filling the gap, and providing tensile strength.

### 2.3 HDAC Inhibition Enriches Immune Response and Tissue Regenerating Genes

**Figure 3:**
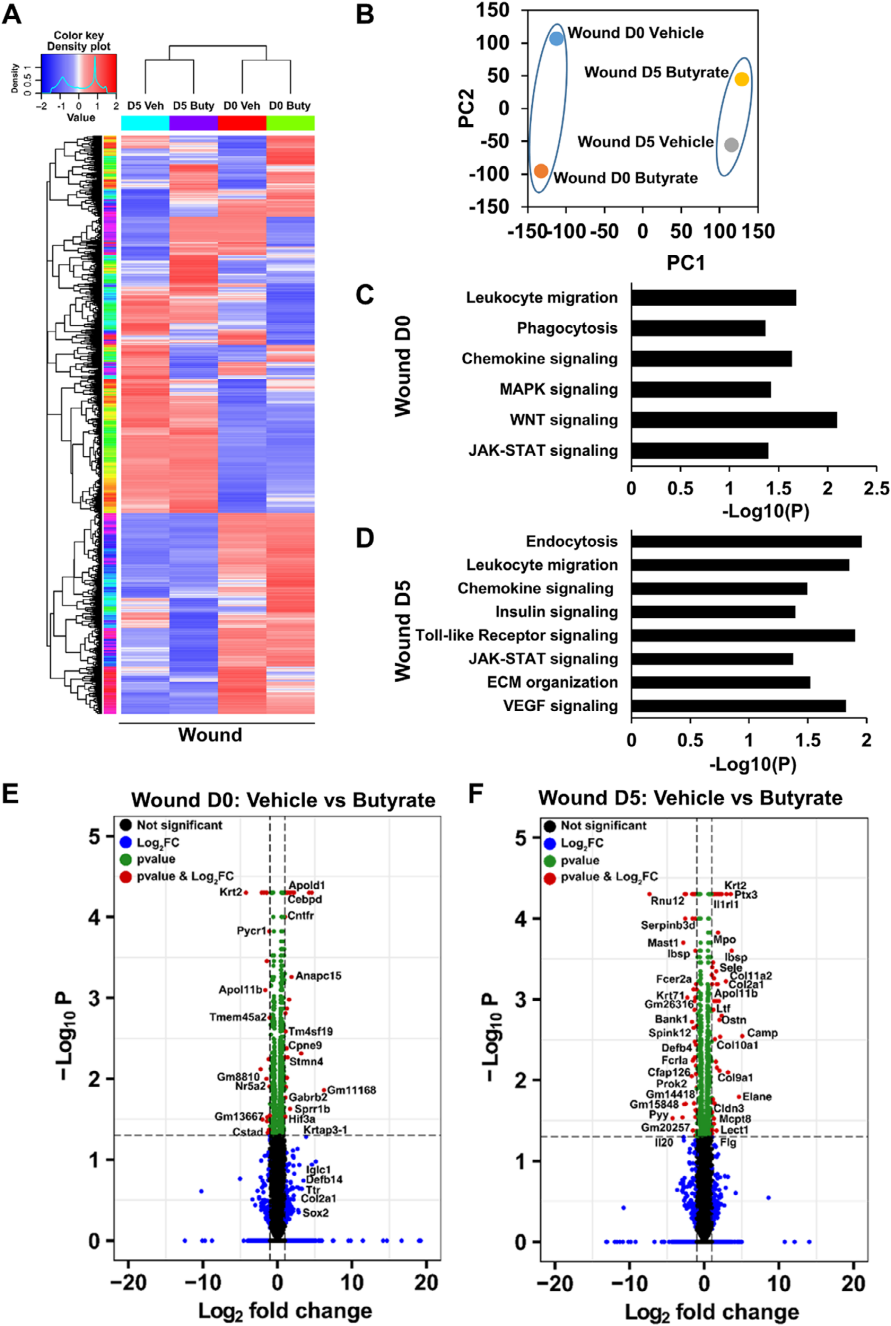
Butyrate rewires transcriptome of normal and wounded skin of diabetic mice. **A)** Heatmap depicting changes at transcriptional level in the skin (D0) of vehicle or butyrate treated diabetic mice and 5 days old wounds (D5). **B)** PCA plot from vehicle and butyrate treated mice skin and wounds. **C)** Pathway enrichment analysis demonstrating key signaling pathways, which are upregulated significantly enriched in skin and **D)** day 5 skin wounds of butyrate treated *db/db* mice compared to vehicle group. **E)** Volcano plot showing differentially regulated genes in unwounded skin and **F)** day 5 wounds of butyrate treated *db/db* mice compared to vehicle group. The red dots represents the genes that are differentially expressed based on log_2_FC cutoff ± 1 and p value cutoff 0.05. The green dot represents the genes, which are differentially expressed based only on p value cutoff 0.05 and blue dots represents the genes, which are differentially expressed based only on log_2_FC cutoff ± 1.

Next, we wished to explore how butyrate exerts its beneficial effect on diabetic wound healing. For this purpose, we analyzed the global transcriptome of biopsies collected from uninjured skin (D0) and D5 wounds of *db/db* mice that either were treated with the vehicle or butyrate (**Figure** **3**A,B). Employing RNA seq analysis and bioinformatic tools, we investigated the pathways activated by butyrate in uninjured skin (Figure 3C) and D5 wounds (Figure 3D) of *db/db* mice. We ranked all genes by their relative enrichment in either normal skin or wounds, followed by functional classification of the most characteristic genes according to Kyoto Encyclopedia of Genes and Genomes database (KEGG) pathways (Figure 3C,D).

Our analysis of unwounded skin (D0) of mice that received butyrate for 7 days revealed differential gene expression and strong enrichment of pathways related to JAK‐STAT, WNT, and MAPK pathways as compared to vehicle (Figure 3A,C). In addition, gene clusters were up regulated, which are associated with higher immune cell activity, including chemokine signaling, phagocytosis, and leukocyte transendothelial migration (Figure 3A,C).

Of note, many of these pathways were also shared by wounded skin (D5) from butyrate‐treated diabetic mice (Figure 3A,D). As opposed to the vehicle group, butyrate‐treated day 5 wounds revealed a profound enrichment of genes associated with JAK‐STAT, Toll‐like receptor signaling, and insulin signaling as well as strong expression of gene sets associated with higher immune cell or macrophage activity, including chemokine signaling, leukocyte transendothelial migration, and endocytosis (Figure 3A,D). The enriched gene signatures following butyrate administration are indicative of a highly active pathway initiated by strong immune responses related to JAK‐STAT and other molecular regulators. Consistent with pathway analyses, volcano plot analysis displayed enhanced expression of genes involved in immune regulation, such as Cebpd, Defb14, TM4SF19, Ptx3, Ilrl1, Mpo, and Elane in butyrate treated skin (D0) and wounds (D5) of *db/db* mice (Figure 3E,F). Butyrate not only enhanced immune response genes, but also promoted the expression of keratinocyte (Krtap 3‐1, Krt2, Flg) and fibroblasts (Col2α1, Col11α2, Col10α1, and Col9α1) specific genes (Figure 3E,F), which are essential for effective tissue repair. These findings are in line with our earlier results showing enhanced matrix deposition in butyrate‐treated diabetic wounds (Figure 2H). Together, these transcriptomic data highlight the benefits of butyrate administration to efficiently promote a beneficial immune response, collectively enforcing wound healing in diabetic mice.

### 2.4 Butyrate Promotes Macrophage Recruitment and Activity in Diabetic Mice Wounds

**Figure 4:**
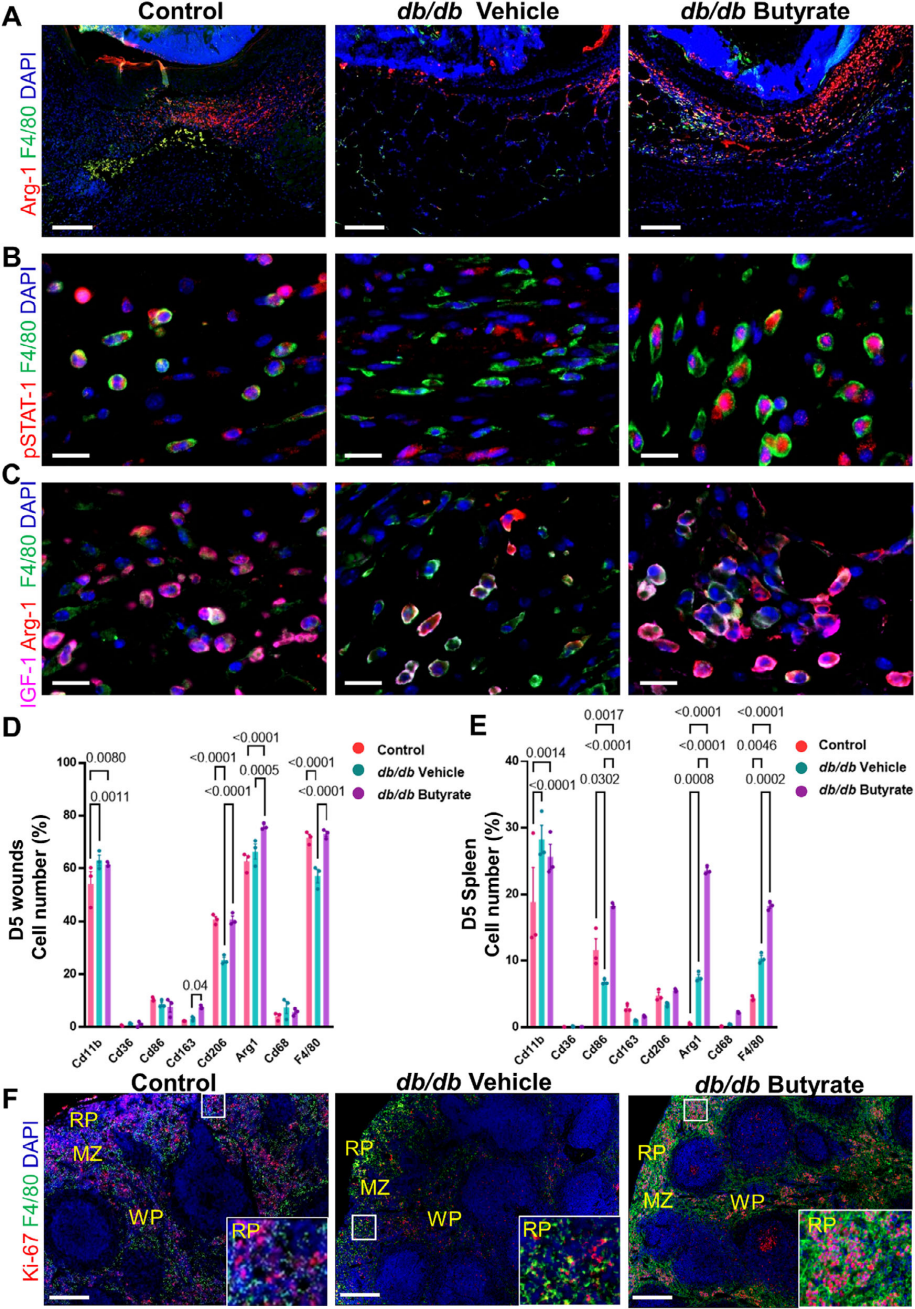
Butyrate facilitates mobilization and activation of tissue resolving macrophages at the wound site in diabetic mice. A) Representative immunofluorescence microphotographs of Arg‐1 (red) stained tissue resolving macrophages and pan macrophage marker F4/80 (green) in 5 days wound sections of either control, butyrate or vehicle treated *db/db* mice and their quantification. Cell nuclei are stained with DAPI (blue). Scale bar, 200 µm. **B)** Representative immunofluorescence microphotographs of pSTAT1 (red) indicative of activated macrophages and the pan macrophage marker F4/80 (green) in 5 day wound sections of either control, butyrate or vehicle treated *db/db* mice. Cell nuclei are stained with DAPI (blue). Scale bar, 20 µm. **C)** Representative immunofluorescence microphotographs of IGF‐1 (magenta), Arg‐1 (red) indicative of tissue resolving macrophages and pan macrophage marker F4/80 (green) in 5 day wound sections of either control, butyrate or vehicle treated *db/db* mice. Cell nuclei are stained with DAPI (blue). Scale bar, 20 µm. **D)** Flow cytometry analysis of the indicated macrophage populations from total cells isolated from D5 wounds by enzymatic digestion. All p‐values were obtained from unpaired t‐test, values are represented as mean ± SEM, *n* = 3. **E)** Quantification of indicated macrophage populations through flow cytometry, cells were isolated by mechanical disruption of whole spleen (D5). All p‐values were obtained from One‐way ANOVA, values are represented as mean ± SEM, *n* = 3. **F)** Representative immunofluorescence staining of microphotographs of Ki‐67 stained (red) proliferating cells and pan macrophage marker F4/80 (green) in spleen sections (D5) of either control, butyrate or vehicle treated *db/db* mice. Cell nuclei are stained with DAPI (blue). Red pulp (RP) and white pulp (WP); in between these two regions is the marginal zone (MZ) in rodent spleen. Scale bar, 200 µm.

Given the marked granulation tissue formation and enrichment of pathways indicating higher immune cell activity in wounds of butyrate‐treated mice, we next studied macrophages as key cells enforcing granulation tissue formation. Immunostaining of key macrophage markers like Arg‐1 and F4/80 revealed significant recruitment of these macrophages to the site of injury in butyrate‐treated *db/db* mice as compared to the vehicle‐treated *db/db* group (Figure 4A; Figure S6A, Supporting Information). Thus, this finding confirms our observations at the transcriptional level, overall suggesting enhanced macrophage activity and an increased transendothelial migration by butyrate. Healthy control mice known to mount a strong inflammatory response, also displayed enhanced macrophage recruitment (**Figure** **4**A; Figure S6A, Supporting Information).

Furthermore, similar to control butyrate significantly increased the expression of pSTAT1 in wound site macrophages, indicating their activation with a regenerative transcriptome signature (Figure 4B; Figure S6B, Supporting Information). Regenerative macrophages are endowed with the unique capacity of enhanced growth factors release enforcing growth and proliferation at the wound site. Indeed, analysis of IGF‐1 co‐expressing macrophages uncovered an increase in IGF‐1 expression in macrophages at the wound site in butyrate‐treated *db/db* mice as opposed to reduced IGF‐1 staining in macrophages from vehicle‐treated *db/db* mice (Figure 4C; Figure S6C, Supporting Information). Butyrate‐mediated IGF‐1 expression in macrophages was comparable to control wounds (Figure 4C; Figure S6C, Supporting Information).

Employing complementary FACS analysis, we further analyzed macrophages in a cell suspension prepared by enzymatic digestion of D5 wounds from control, vehicle, and butyrate‐treated diabetic mice. FACS analysis shows that a key macrophage population, including (Cd163, Cd206, Arg‐1 and F4/80) were significantly increased in wound samples from butyrate as compared to vehicle‐treated wounds (Figure 4D). These data suggest that regenerative macrophages—likely by better recruitment or differentiation—occur at higher numbers at the diabetic wound site after butyrate administration.

The spleen acts as a reservoir for undifferentiated monocytes that readily mobilize to other organs under inflammatory or trauma conditions.^[^ ^38^, ^39^ ^]^ We here set out to analyze the spleen as a prime organ, where monocytes transform into macrophages endowed with phagocytic activity. FACS analysis of cell suspensions prepared from the spleen of diabetic mice with day 5 wounds revealed that several macrophage populations, including Cd86, Arg‐1, and F4/80 were increased upon butyrate administration as opposed to the vehicle group (Figure 4E). The immunophenotype of wounds and spleens from diabetic mice treated with butyrate showed many characteristics like those of healthy controls (Figure 4E,F). Like in skin wounds, butyrate expanded macrophages (F4/80) in the red pulp and white pulp of the spleen (Figure 4F; Figure S6D,E, Supporting Information). This increased expansion of the macrophage compartment in the spleen nicely correlates with an enhanced number of wound macrophages following butyrate treatment (Figure 4A). This increase in macrophage subsets by butyrate most likely accelerated diabetic wound repair, that typically suffered from weak inflammatory response.

### 2.5 Single‐cell Atlas Reveals Butyrate‐Induced Wound Remodelling in Diabetic Mice

Next, we aimed to decode the specific impact of butyrate administration in shaping macrophages dynamics and their interaction with distinct cell lineages during skin regeneration in *db/db* diabetic mice. Therefore, we generated a single cell atlas from un‐injured skin (D0) and wounds (D5 and D10) of healthy non‐diabetic control and diabetic mice treated either with vehicle or butyrate.

**Figure 5:**
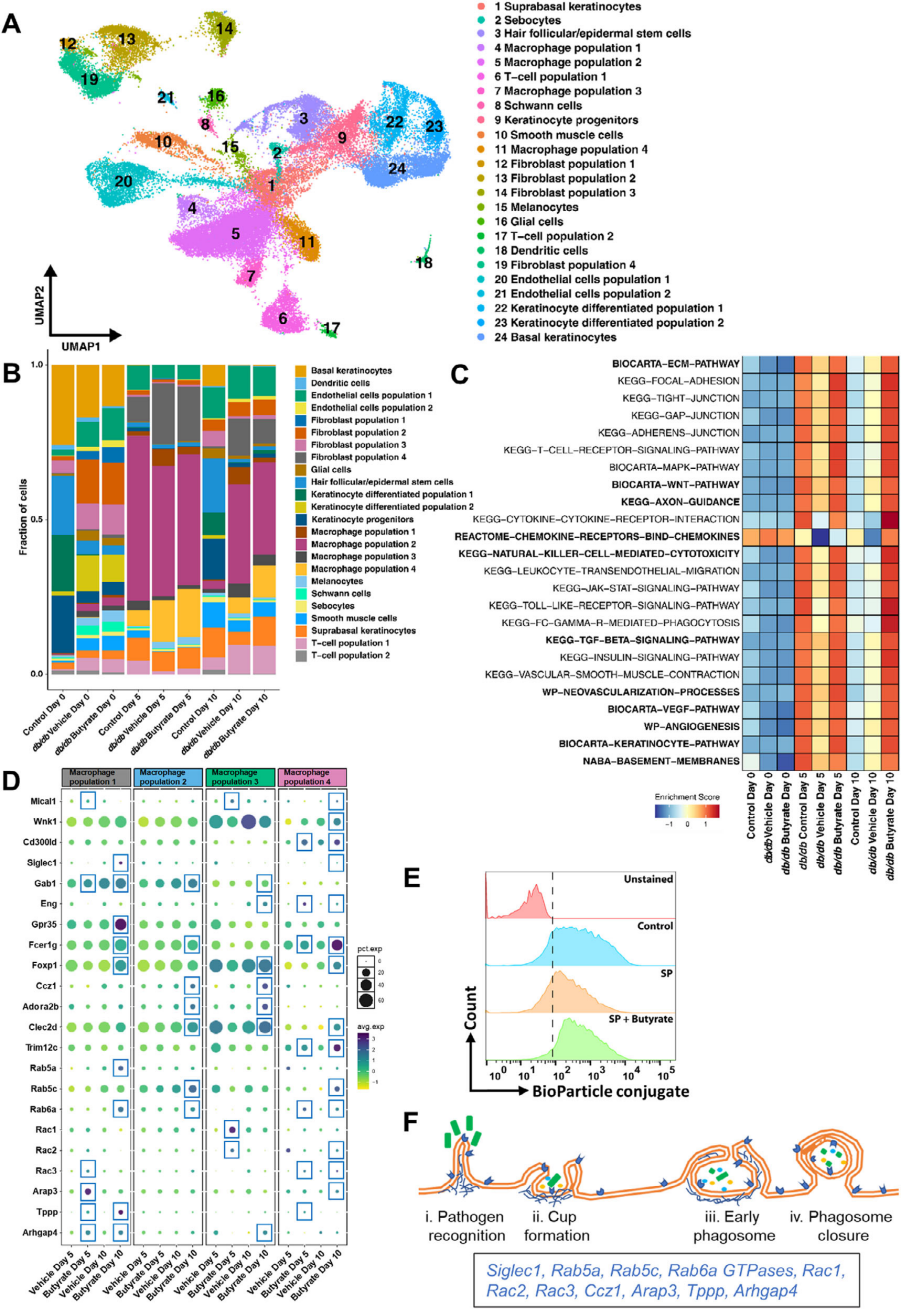
Butyrate reprograms the single‐cell transcriptome in diabetic wounds. **A)** Uniform Manifold Approximation and Projection (UMAP) visualization of dissociated diabetic mice wound cells from entire dataset consisting of 48 000 cells. The broad annotation of cell states is denoted by color and number in the legend. **B)** Stacked bar plots showing average proportions of skin cells at indicated time points from control, vehicle and butyrate treated diabetic mice during normal physiology and repair. Different cell states are denoted by color in the legend. **C)** Selected biological pathways that are significantly (*p* < 0.01) affected in control and butyrate administrated *db/db* mice as compared to vehicle treated *db/db* mice. **D)** Dot plot showing variance‐scaled, percent of cells expressing (dot size) genes and mean expression (dot color) in four different macrophages subpopulation of *db/db* mice wounds. Red square labels indicate genes upregulated by butyrate. **E)** FACS analysis displaying the phagocytotic uptake of pHrodo Red *E. coli* bacteria by macrophages from distinct treatment groups for 60 min at 37 °C. An increase in mean fluorescence intensity indicating the active uptake of *E. coli* by macrophages. **F)** Schematic outline of different stages of phagocytosis driven by butyrate activated genes as indicated in the rectangle below.

Our scRNA‐seq dataset from skin wounds comprised 48 000 cells, which passed the quality control. Broad cell labels as for erythroid cells, keratinocytes, hair follicle/epidermal stem cells, endothelial cells, macrophages, fibroblasts, T‐cells, dendritic cells, smooth muscle cells, glial cells, sebocytes, granulocytes, and their subtypes were assigned (**Figure** **5**A) based on differentially expressed genes (DEGs).^[^ ^12^, ^40^ ^]^

Differential abundance analysis revealed a dynamic shift in cell populations physiologically maintaining skin homeostasis versus those participating in tissue repair (Figure 5B). Control and diabetic wounds revealed marked differences in population dynamics both during tissue repair (D5 and D10) and under normal physiology (D0) (Figure 5B). Tissue repair process in control involves a balanced participation of tissue resident cells and immune cells, whereas diabetic tissue repair was dominated by macrophages (Figure 5B). Another notable difference between healthy and diabetic skin was that at the later stages of repair, most of the cellular components were restored in healthy skin compared to diabetic skin (Figure 5B). As opposed to the vehicle control, butyrate administration altered the cellular composition of both non‐injured skin and injured skin undergoing repair in diabetic mice (Figure 5B). For example, “macrophage population 4′, ‘suprabasal keratinocytes” and ‘smooth muscle cell population 1′ were increased, while ‘macrophage population 1′ was decreased in the wounds of butyrate‐treated *db/db* mice as compared to the vehicle group (Figure 5B).

Since the number of macrophages and their populations alone does not reflect their activity or functional state, we further explored the transcriptomic network. Consistent with our bulk RNAseq results (Figure 3A–F), several pathways facilitating tissue repair were significantly upregulated in diabetic mice group that received butyrate (Figure 5C). The pathways regulating ‘cell‐cell interaction’, “cell migration”, “pathogen clearance”, “regulation of immune activity and inflammation”, “cellular proliferation and differentiation”, “angiogenesis”, “formation of tight junction”, and “the synthesis of extracellular matrix” during skin repair were overrepresented in the control and butyrate group compared to the vehicle group. (Figure 5C). Similar to control wounds, most of these pro‐repair pathways were activated quite early (D5) in butyrate‐treated *db/db* mice as compared to the vehicle group (Figure 5C).

Macrophage recruitment and phagocytosis play a pivotal role in the resolution of inflammation at the wound site and is severely disturbed in diabetic wounds.^[^ ^7^ ^]^ In fact, in butyrate group, we observed an overrepresentation of the Mical1 gene coding for a monooxygenase, which—via reactive oxygen species—enhances migration^[^ ^41^ ^]^ of ‘D5 macrophage population 1 and 3’ (Figure 5D). In addition, the Eng1 gene coding for CD105 cell surface protein,^[^ ^42^ ^]^ enforces macrophage migration in ‘D5 macrophage population 4’ (Figure 5D), and of the Cxcl1 gene coding for a chemokine recruiting monocytes^[^ ^43^ ^]^ in ‘D5 macrophage population 1’ of butyrate‐treated *db/db* mice (Figure S7A, Supporting Information).

We found an activation of gene programs regulating different stages of phagocytosis (Siglec1,^[^ ^44^ ^]^ Rab5c,^[^ ^45^ ^]^ Rab6a,^[^ ^46^ ^]^ Ccz1,^[^ ^47^ ^]^ Arap3,^[^ ^48^ ^]^ Tppp ^[^ ^49^ ^]^ and Arhgap4^[^ ^50^ ^]^) in macrophage subsets from butyrate‐treated wounds (Figure 5D). To functionally validate these transcriptomic results, we studied phagocytosis of macrophages using fluorescently labelled *E. coli* bacteria. The FACS and fluorescence microscopy analysis depicted a profound decrease in phagocytic activity of macrophages pre‐exposed with SP (Figure 5E; Figure S7B, Supporting Information). HDAC inhibition by butyrate significantly restored the phagocytic functions of macrophages exposed to the SP (Figure 5E; Figure S7B, Supporting Information). Together, these findings indicate that butyrate facilitate different steps of phagocytosis by macrophages under diabetic conditions (Figure 5F).

As to inflammation control, we made the interesting observation that the dichotomy of pro‐inflammatory macrophages versus anti‐inflammatory macrophages does not apply for wound healing of butyrate treated diabetic mice, we even found that at day 10 macrophage population 4 highly express Trim12c, a pro‐inflammatory ubiquitin ligase (Figure 5D), which stimulates type I interferons and NF‐ĸB, important in infection control by the innate immune system.^[^ ^51^ ^]^ By contrast, the transcription factor Foxp1, which suppresses cytokine production (TNF‐α, IL‐6, IL1β), phagocytosis, and respiratory burst^[^ ^52^ ^]^ was significantly increased in macrophages in day 10 wounds of butyrate‐treated *db/db* mice (Figure 5D), suggesting coexistence of hybrid macrophages. Though classical M1 and M2 macrophage populations^[^ ^53^ ^]^ differentiated under in vitro conditions expresses unique set of markers (Figure S8A, Supporting Information, derived from publicly available data sets^[^ ^54^ ^]^). However, this in vitro classification involving specific factors is an oversimplification of the highly complex and dynamic state of macrophages in vivo. Overall, butyrate tilts the response towards anti‐inflammation by enhancing phagocytosis, and a clear reduction of mRNA coding for detrimental pro‐inflammatory cytokines like IL‐1β, IL‐6, Litaf, and TNF‐α (Figure S7A, Supporting Information). We further confirmed butyrate‐mediated suppression of IL‐1β and TNF‐α at the protein level (Figure S8B–E, Supporting Information). IL‐1β is highly increased in chronic wounds among them diabetic wounds driving the non‐healing state of these wounds.^[^ ^14^, ^55^ ^]^ TNF‐α and Litaf activates pro‐inflammatory macrophages and keep them in a vicious cycle of unrestrained activation.^[^ ^56^ ^]^ Notably, CD36, a receptor responding to fatty acid and pathogen‐associated molecular patterns,^[^ ^57^ ^]^ contribute to inflammation were down regulated by butyrate in wound macrophages (Figure S7A, Supporting Information). In addition, genes like Fcer1g^[^ ^58^ ^]^ and Gab1, enhancing anti‐inflammatory IL‐4 levels in macrophages^[^ ^59^ ^]^ were upregulated in different macrophage population’ of butyrate treated *db/db* mice wounds (Figure 5D). Genes were enriched in butyrate treated *db/db* mice, which directly protect the integrity of the macrophages including the Cd300id gene responsible for immune suppression of myeloid cell‐derived suppressor cells^[^ ^60^ ^]^ and the Clec2d gene suppressing cell cytotoxicity of natural killer cells^[^ ^61^ ^]^ in ‘D5 and D10 macrophage population 4’, while autophagy gene Atg4a was downregulated in all four macrophage type (Figure 5D; Figure S7A, Supporting Information).

Moreover, Reactome/GSEA analysis algorithms were employed to analyse the epigenetic transcriptome of wound macrophages and to find epigenetic adaptation during the healing process in vivo. we observed a differential regulation of epigenetic mediators during wound repair, for example ‘macrophage population 2’ revealed general suppression, while upregulation of genes in ‘macrophage population 4’ was observed upon injury as compared to uninjured skin (Figure S9A,B, Supporting Information). These results indicate an active role of “macrophage population 4” during tissue repair, and this view was further supported by the fact that they were strongly expanded by butyrate (Figure 5B), suggesting a central role for this ‘macrophage population 4’ in promoting diabetic wound healing. Butyrate administration, irrespective of macrophage subtype showed a strong alteration of epigenetic regulators in diabetic mice wounds (Figure S9A,B, Supporting Information). These findings highlight epigenome remodelling by butyrate, which broadly promotes tissue repair in a diabetic microenvironment.

Collectively, we herein identified that macrophage activation in wounds of butyrate‐treated *db/db* mice shift their transcriptomics to a clear reduction of chronic inflammation by up‐regulating phagocytosis and through a concerted balance of necessary local inflammatory signals and anti‐inflammatory control to promote repair. These paracrine factors released by macrophages may have an enormous impact on distinct keratinocyte populations and their progeny as well as on distinct cell populations of dermal tissue.

### 2.6 Butyrate Activates Key Repair Pathways and Crosstalk Between Macrophages, Keratinocytes, and Fibroblasts in Murine Diabetic Wounds

**Figure 6:**
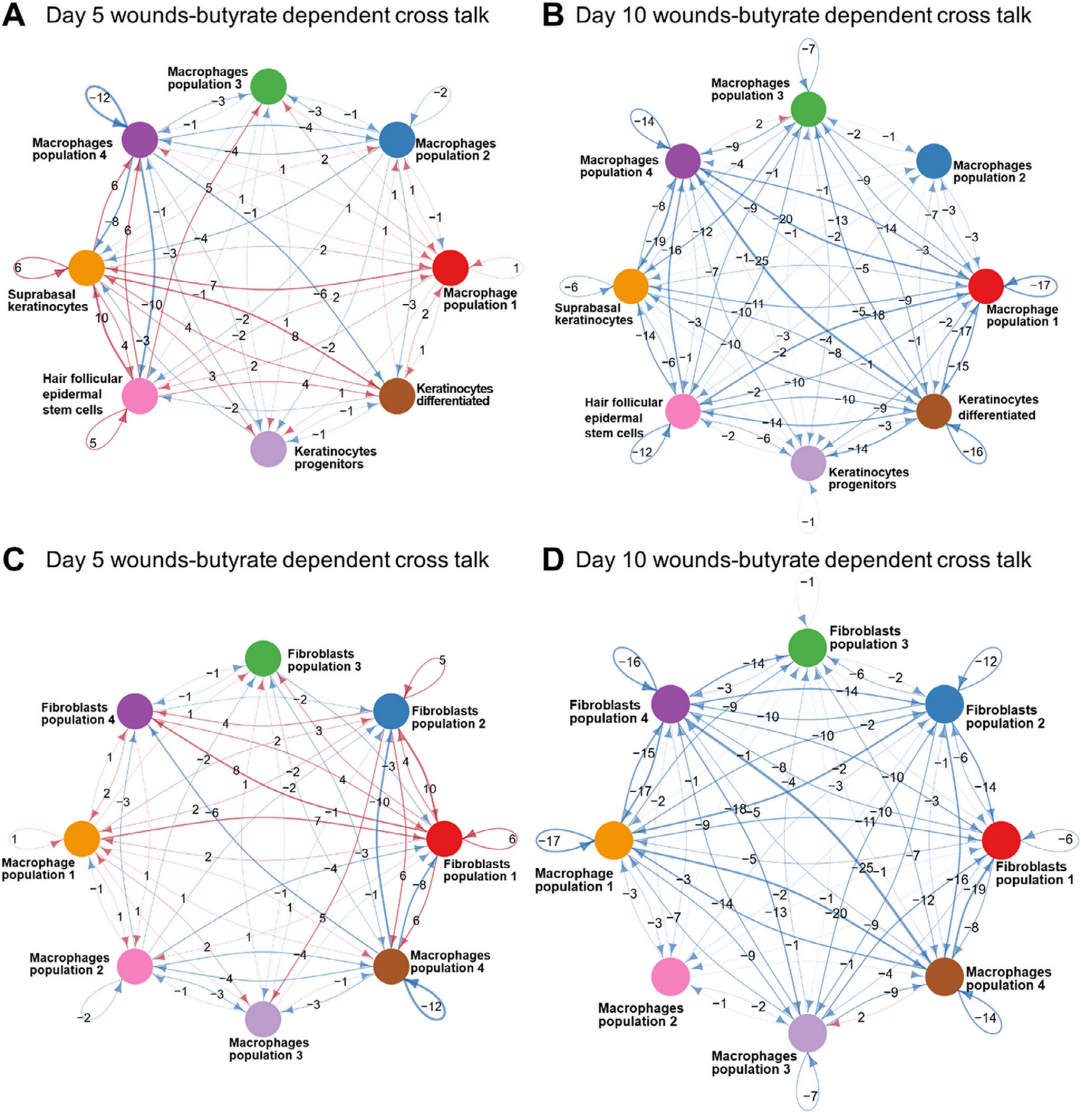
Butyrate reprograms different macrophage populations and the cross talk with other wound cells overcoming the non‐healing state of diabetic wounds. **A)** Circular plot of cell‐cell cross talk between different subsets of macrophages and keratinocytes in D5 and **B)** D10 wounds. Intensity of interactions of different macrophage populations with different keratinocyte subsets, indicated by red (high) and blue (low) lines is induced in wounds of butyrate treated diabetic mice when compared to wounds of vehicle treated diabetic mice. The line thickness represents number of interactions. **C)** Circular plot of cell‐cell cross talk between different subsets of macrophages and fibroblasts in D5 and **D)** D10 wounds. Significant cell‐to‐cell interactions, indicated by red (high) and blue (low) lines, are induced by butyrate in diabetic wounds compared to vehicle. The line thickness represents number of interactions.

The reconstitution of the epidermal barrier following tissue injury depends on keratinocyte migration, proliferation, and differentiation.^[^ ^62^ ^]^ Keratinocytes migration is severely impacted by diabetes,^[^ ^10^ ^]^ most likely due to impaired communication with other cells participating in wound repair. To address this, CellChat was used to analyse the cell‐to‐cell communication between keratinocytes and macrophages during wound healing. Butyrate administration enhanced the interaction of several macrophage populations with keratinocytes and their stem cell derivatives at the early phase of wound healing (D5) as opposed to vehicle‐treated diabetic wounds (**Figure** **6**A), highlighting a faster activation and early participation of keratinocytes during re‐epithelialisation and tissue repair. Our findings are in line with an earlier report where healers versus non‐healers of human diabetic foot ulcers (DFU) were analysed.^[^ ^12^ ^]^ Enrichment of macrophages overexpressing inflammatory genes, including IL‐1β, S100A8, and S100A9 in DFU‐healers was observed to stimulate an acute inflammatory response to promote wound healing (Figure S9C,D, Supporting Information, derived from publicly available data sets ^[^ ^12^ ^]^).

This crosstalk between macrophages and keratinocytes was reduced at later stages of repair in D10 wounds of butyrate treated *db/db* mice as opposed to wounds of vehicle‐treated *db/db* mice (Figure 6B).

Furthermore, heatmap analysis of different cell types depicted that diabetic wound treated with butyrate adopts a faster healing kinetics compared to the slow and inefficient repair in the vehicle (Figure S10A,B, Supporting Information). Apart from butyrate‐activated macrophages and their interaction with other wound cells, we cannot rule out a direct effect of butyrate on other cell populations, including fibroblasts, keratinocytes, and endothelial cells at the wound site.

Higher expression of Keratin 10 was observed in differentiated keratinocytes, indicating induction of differentiation and restoration of the epidermis and skin integrity by butyrate treated wounds (Figure S11A, Supporting Information). Butyrate treated wound showed induction of the pioneer factor Sox9,^[^ ^63^, ^64^ ^]^ which drives the activation of hair follicle stem cells supporting self‐renewal and epidermal homeostasis in *db/db* mice skin (Figure S11A, Supporting Information). These data support the view of strong interactions between macrophage subpopulations and distinct epidermal cell populations (Figure 6A) with an enhanced reconstitution and maintenance of the epidermal barrier.

Dermal fibroblasts are also essential for tissue repair and skin homeostasis, and during injury a complex interrelationship between macrophages and fibroblasts has been supposed though not fully elucidated.^[^ ^65^, ^66^ ^]^ Single cell analysis depicted 4 fibroblast populations (Figure S11B, Supporting Information). In fact, along with other growth factors, IGF‐1 expression was induced in ‘D10 fibroblast subpopulation 4’ in butyrate treated *db/db* mice that facilitates growth and repair of wounds (Figure S11B, Supporting Information). Interestingly, both ‘D5 fibroblast population 3 and ’D10 fibroblast population 4′ revealed higher expression of Tgf3 coding for TGF‐β3 (Figure S11B, Supporting Information), an antifibrotic molecule which prevents over healing and the formation of hypertrophic scars.^[^ ^67^ ^]^ Cell‐to cell interaction graphs from CellChat demonstrated robust interaction between macrophage subpopulations with fibroblasts in butyrate treated *db/db* wounds, which were initiated at early and readily diminished during late phase of tissue repair (Figure 6C,D). These interactions with different fibroblast populations likely contribute to the observed acceleration of wound healing in butyrate‐treated *db/db* mice (Figure 2G).

Collectively, butyrate induced epigenetic changes with suppression of HDAC in diabetic macrophages switch their transcriptome towards enhanced migration, phagocytosis, and tissue resolution with the concomitant release of growth factors. Together, these series of harmonized events initiate a paracrine crosstalk with fibroblasts and epidermal cell populations to favour diabetic tissue repair.

### 2.7 Butyrate Suppresses the Vicious Inflammatory Cycle in Non‐Healing Wounds

**Figure 7:**
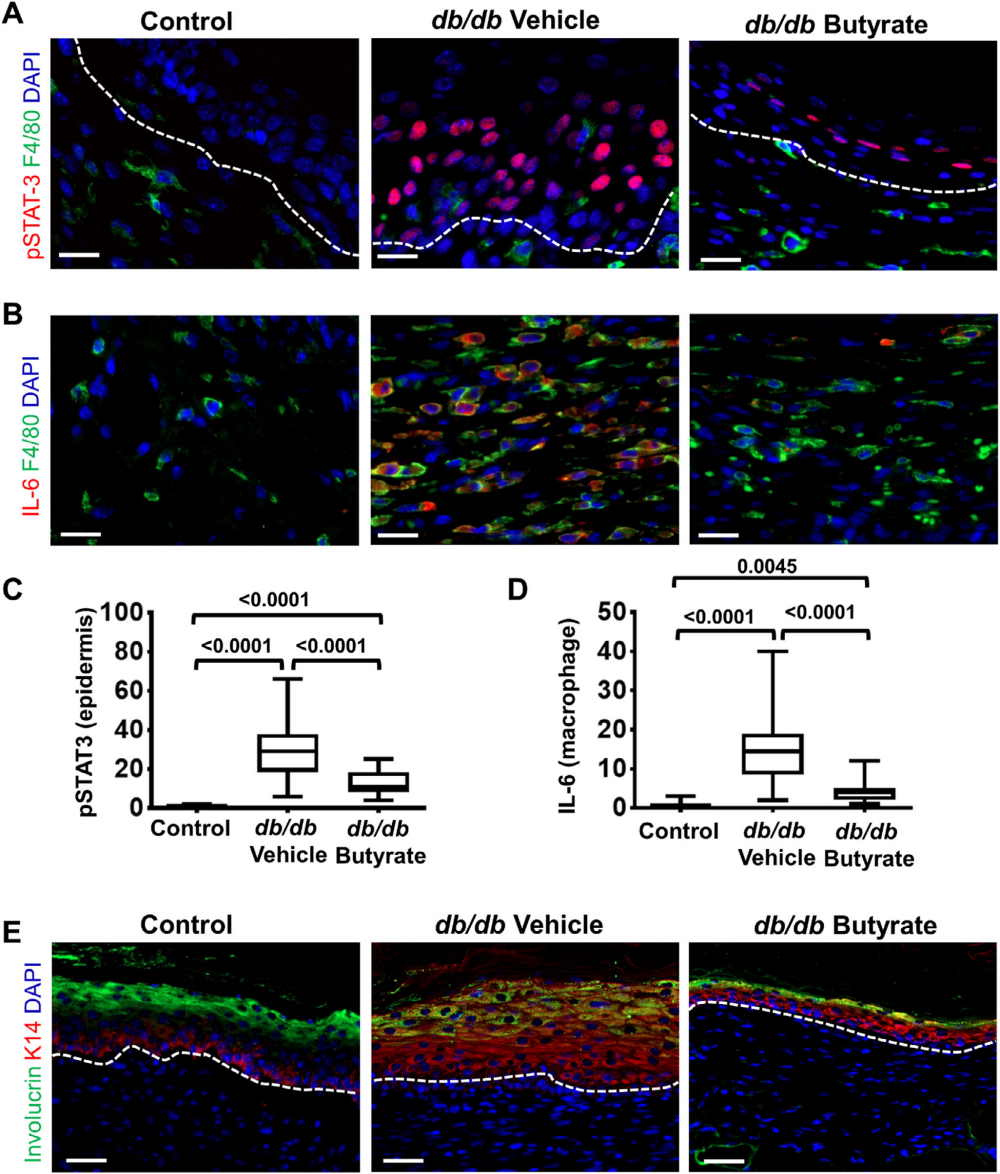
Butyrate restricts induction of persistent inflammation in diabetic wounds. A) Representative microphotographs with immunostaining of pSTAT3 (red) indicative for the inflamed epidermis and pan macrophage marker F4/80 (green) in 10 days old wound sections of either control, butyrate or vehicle treated *db/db* mice. Nuclei stained with DAPI in blue. Scale bars, 20 µm. **B)** Representative immunostaining microphotographs of IL‐6 (red) and pan macrophage marker F4/80 (green) in 10 days old wound sections of either control, butyrate or vehicle treated *db/db* mice and their quantification. Cell nuclei are stained with DAPI (blue). Scale bar, 20 µm. **C)** Quantification of the pSTAT3 positive keratinocytes of wounded epidermis presented in (A), One‐way ANOVA values are represented as mean ± SEM, *n* = 5. **D)** Quantification of IL‐6 expressing macrophages presented in (B). One‐way ANOVA, values are represented as mean ± SEM, *n* = 5. **E)** Representative microphotographs with immunostaining of differentiated keratinocytes marker involucrin (green) and undifferentiated keratinocytes marker K14 (red) in 10 days old wound section from control, butyrate treated *db/db* mice compared to vehicle treated group (*n* = 5). Nuclei stained with DAPI in blue. Scale bars, 20 µm.

To further elucidate the outcome of macrophage activation and their crosstalk with keratinocytes on epidermal homeostasis, we determined the role of butyrate in obesity and diabetes induced inflammation. Enhanced protein expression of inflammatory marker pSTAT3^[^ ^68^, ^69^ ^]^ was observed in almost all nuclei of keratinocytes within the hyperplastic epidermis (Figure 7A) and strong IL‐6 staining occurred in macrophages associated to adipocytes in day 10 wounds of vehicle‐treated *db/db* mice (Figure 7A–D). Repetitive administration of butyrate suppresses IL‐6 expression in macrophages (Figure 7B,D) and reduced the paracrine pSTAT3 induction in the epidermis (Figure 7A,C), and this breaks the cycle of unrestrained inflammation. By contrast, control wounds did not show any expression of both these proinflammatory markers pSTAT3 and IL‐6 (Figure 7A–D). Butyrate potentially improves macrophage function and thus diabetic‐associated wounds by repairing histone acetylation defects (Figure S11C,D, Supporting Information). This IL‐6 expression may partly be driven by the activation of cJUN in macrophages under diabetic condition SP (Figure S11E,F, Supporting Information). Of note, an upregulation of activated pcJUN and its downstream target gene IL‐6 was detected in macrophages following exposure to SP (Figure S11E, Supporting Information). The increase in pcJUN and IL‐6 was remarkably suppressed to normal levels by the HDAC‐inhibitor butyrate in SP‐exposed macrophages (Figure S11E, Supporting Information).

Furthermore, immunostaining of the late differentiation keratinocyte marker involucrin and the undifferentiated K14^[^ ^70^ ^]^ in day 10 wounds from vehicle‐treated *db/db* mice revealed an expansion of epidermal cell layers indicative of epidermal hyperplasia (Figure 7E). Vehicle‐treated diabetic wounds showed diffuse expression of involucrin in the upper epidermal layers and K14 in the lower layers, key changes typically occurring in inflamed epidermis (Figure 7E). Like in control wounds, in diabetic wounds butyrate restricted this inflammation‐induced hyperplasia to 2 to 3 epidermal layers and balanced the expression of differentiation markers to that of a fully restored normal epidermis morphology with clear demarcation of upper differentiated and lower undifferentiated layers (Figure 7E). Butyrate synergistically dampens persistent inflammation and induces re‐epithelialization in non‐healing diabetic wounds and thereby enhances tissue repair. These findings are consistent with our single cell results from three keratinocytes subpopulations indicating higher expression of differentiation genes and reduced expression of inflammatory mediators in keratinocyte populations upon butyrate treatment (Figure S11A, Supporting Information).

In conclusion, our findings demonstrate a loss of acetyl histone‐H3 (Lys27) mark and suppression of the dominant transcription factor STAT1 in macrophages under diabetic conditions. Inhibition of HDACs by butyrate corrects these defects in vivo, generating a rapid and effective macrophage‐mediated innate immune response in diabetic wounds, thus facilitating a smooth transition to the subsequent growth and remodeling phase of tissue repair. Butyrate not only restores epigenetic and transcriptional control but also rebalances the temporal and spatial activity of macrophages. Comprehensive remodeling of macrophage biology through butyrate endows them with the ability to better coordinate other participating cells of tissue repair, eventually leading to improved healing of diabetic wounds.

## 3 Discussion

**Figure 8:**
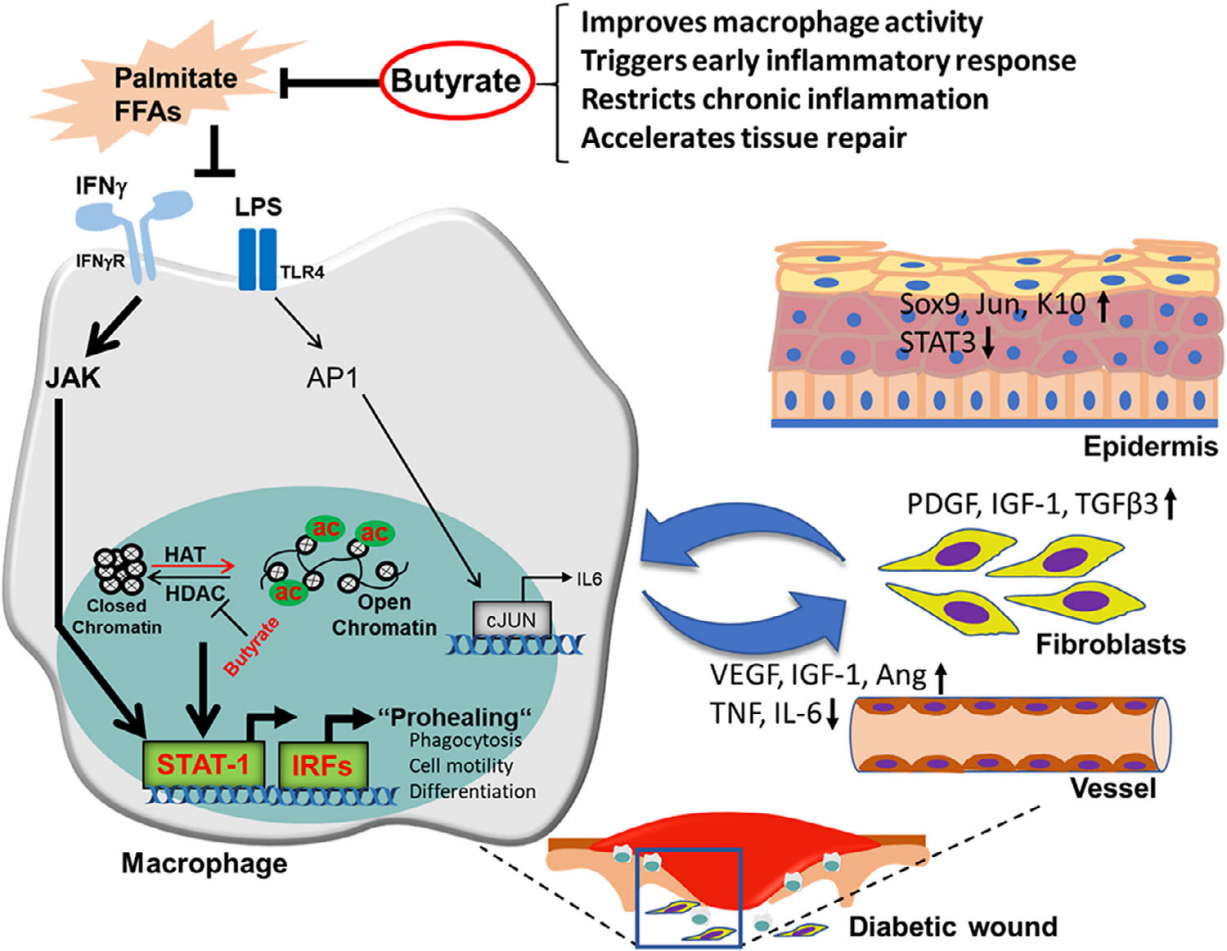
Graphical summary Cartoon summarizing benefits of HDAC inhibition in macrophages residing in the pro‐inflammatory diabetic microenvironment. Comprehensive transcriptomic analysis identified functional impairment of diabetic macrophages with suppression of active histone marks (acetyl histone‐H3). The HDAC inhibitor butyrate led to the restoration of histone acetylation resulting in reprogramming the transcriptome and gene networks in macrophages enforcing improved healing. Butyrate induced reprogrammed macrophages exhibit a unique phenotype with rapid recruitment, enhanced early inflammatory response and phagocytic activity at the wound site of diabetic mice. Additionally, butyrate enhanced macrophage interactions with other cells including keratinocyte and fibroblast subpopulations fostering re‐epithelialization, angiogenesis and filling the wound gap by matrix deposition. Together, this more rapidly initiates a tissue repair program in early stages of wound healing and also prevents chronic inflammation associated with chronic diabetic ulcers.

Our major finding highlights the loss of a specific acetylation mark of histone core as a prime driver for abrogated macrophage function during tissue repair in diabetic mice and its attenuation by butyrate, a pharmacologic inhibitor of the histone acetylase (Figure 8, graphical summary).

Unbiased comprehensive transcriptome analysis employing bulk and single cell sequencing of wound tissues with complementing functional analysis, identified that the epigenome pathways regulating innate immune cells like macrophages are strongly affected in diabetic mice. Diabetic conditions not only suppress the acetylated histone mark in macrophages but also dysregulate their major inflammatory signaling pathways, cytokine profile, phagocytic functions, and the crosstalk with other cells at the wound site. Diabetes related lipotoxicity disrupts the fine‐tuned transcriptional network of macrophages and, in consequence, impairs their differentiation, expansion, and migration towards sites of injury resulting in poor healing. Restoration of the histone mark through pharmacological inhibition of the HDAC by butyrate rescued macrophage activity, induced their phagocytotic capacity, and their crosstalk with other cells at the wound site, eventually contributing to efficient inflammatory response and improved healing of otherwise non‐healing wounds in diabetic mice (Figure 8, graphical summary).

Butyrate is beneficial in several ways for the treatment of delayed healing of diabetic wounds, which is mainly caused by macrophage dysfunction. First, repetitive administration of butyrate enhances histone h3 acetylation (Lys 27) in diabetic macrophages, fostering the transcription of specific wound healing promoting genes. This is accomplished by the opening of chromatin, a prerequisite for the binding of distinct transcription factors such as STAT1 and other family members. Secondly, under diabetic conditions, butyrate activates STAT1 signaling in macrophages, a key regulatory transcription factor that, through activation of downstream target genes and signaling cascades including IRFs, stimulates the phagocytic capacity of macrophages, essentially required to clean wounds from debris and microbes.

Thirdly, butyrate administration enhanced macrophage numbers and, after a cleanup at the wound site, also activates inflammation resolving macrophages and their crosstalk with other cell types, with subsequently enhanced matrix deposition and epidermal restoration at the wound site, all together improving wound healing in diabetic mice. A recent report shows that impaired activity of macrophages and the lack of inflammation‐resolving regenerative macrophages at the site of injury disturbs tissue repair in humans suffering from diabetic foot ulcers.^[^ ^11^, ^12^, ^71^ ^]^ Unexpectedly and previously unreported, we here provide evidence that the HDAC inhibitor butyrate is responsible for the reversal and activation of the suppressed acetylated (Lys27 or Lys9) histone mark in diabetic macrophages promoting the beneficial resolution of the persisting ineffective inflammation of wounds in *db/db* mice. Thereby, butyrate not only activates JAK‐STAT1, chemokines, TLR, and insulin signaling pathways, but also enhances transendothelial leukocyte migration, phagocytosis, and endocytosis.

Finally, butyrate strongly and swiftly activated molecular signaling and thus restricts the entry of wounds into a vicious cycle of persistent inflammation through disruption of the IL‐6 loop in macrophages of diabetic wounds. Inhibition of IL‐6 in turn suppresses STAT‐3 activation in epidermal cells and macrophages at the wound site.

Single cell transcriptomic landscape unveiled the beneficial action of butyrate on macrophages subsets and their crosstalk with other wound cells, promoting wound healing. Recently, the human prenatal skin atlas revealed that the crosstalk between non‐immune and immune cells plays a crucial for hair follicles formation, scarless wound healing, and skin angiogenesis.^[^ ^17^ ^]^

The spotlight of our finding is that butyrate reprograms the transcriptomic signature of macrophages and likely other wound cells under persisting hostile conditions of diabetic mice. This underscores the hierarchy of epigenetic reprogramming over a persisting hostile diabetic microenvironment with high concentrations of saturated free fatty acids. These patients suffering from type 2 diabetes often reveal a profound gut microbial dysbiosis with lower abundance of butyrate producing bacteria.^[^ ^72^ ^]^ We here highlight that butyrate—via epigenetic modulation of the histone core—rebalanced dysregulated macrophage function and promotes wound healing in diabetic mice. In an earlier report butyrate derived from microbiota enhanced the memory potential of activated CD8^+^ T cells.^[^ ^73^ ^]^

In the past, an interrelated interaction between epigenome modifiers and transcriptional regulation and the functionally relevant outcome in pathological conditions have been reported.^[^ ^74^, ^75^, ^76^, ^77^, ^78^ ^]^ Two chromatin modifiers—through lysine monomethylation of STAT1 and IRF1— promote phosphorylation of antiviral signaling transducers in macrophages.^[^ ^74^, ^75^, ^78^ ^]^ Similarly, histone acetylation controls type I interferon receptor and NF‐kB signaling.^[^ ^77^ ^]^ HDAC3 was described to regulate deacetylation of the TGFβ promoter in macrophages ^[^ ^79^ ^]^ and KDM1A demethylates p65 promoting stability of the nuclear p65 protein in macrophages ^[^ ^80^ ^]^ after repetitive insults with inflammatory stimuli.

In conclusion, we provide a comprehensive analysis of murine diabetic wounds with delayed healing and identified reduced levels of acetylated histone core as the prime event causal for disrupted macrophage signalling and poor healing of diabetic wounds. Most importantly, we uncovered butyrate to restore specific histone acetylation with fundamental inflammation control of macrophages and improved wound healing in diabetes.

## 4 Experimental Section

### Macrophage Isolation, Differentiation, and Stimulation

Human monocytes were purified from fresh buffy coats ^[^ ^14^ ^]^ by density gradient using Biocoll separating solution (Millipore) followed by magnetic cell sorting with CD14 micro beads (Miltenyi Biotech). Cells were then cultured in DMEM with 2 mm l‐glutamine, 50 mg mL^−1^ gentamicin (Biochrom), 1 mm sodium pyruvate, and 1 mm non‐essential amino acids at 37 °C, 5% CO_2_, for 48 h. After 48 h, 50 ng mL^−1^ GmCSF was added to allow differentiation of monocytes towards macrophages for 7 days. Following differentiation macrophages were stimulated with the combination 20 ng mL^−1^ IFNγ (PeproTech) and 10 ng mL^−1^ LPS (Sigma‐Aldrich) for 16 h in the presence and absence of 250 µm free fatty acid sodium palmitate, SP (Sigma‐Aldrich) or other indicated pharmacological agents.

### Wound Healing Assay

Wound healing studies were performed as described earlier.^[^ ^81^ ^]^ Briefly, for wound healing experiments, 8 to 10 weeks old male *db/db* diabetic (*BKS.Cg‐Dock7m^+/+^ Lepr^db^/J*) and wildtype control (C57B6/J) mice were used. Before wounding and during wound healing, mice received intraperitoneal injection of either PBS or 500 mg kg^−1^ sodium butyrate on alternate days for 12 days. Mice were directly purchased from Jackson Laboratories and housed under pathogen‐free conditions at the animal care facility of Ulm University in compliance with the German Law for Welfare of Laboratory Animals. All experiments were approved by the RP Tübingen Baden‐Württemberg (approval numbers TVA1289 and TVA1609).

### Western Blotting

Western blot analyses were performed as previously described.^[^ ^82^ ^]^ Briefly, after protein transfer, nitrocellulose membranes were incubated with primary antibodies against pSTAT1, IL‐6 (R&D system), STAT1, IRF‐1, pS6RP, p21, acetyl histone‐H3 (Lys27), acetyl histone‐H3 (Lys9), monomethyl histone‐H3, tri‐methyl histone‐H3 (Lys9), pcJUN, cJUN (Cell Signaling), HDACs (Cell Signaling) and actin (Santa Cruz). Then, the membranes were incubated with HRP‐conjugated secondary antibodies that were purchased from Jackson immune research. The membranes were developed by LumiGLO substrate (Cell Signaling) and visualized by Vilber gel documentation system.

### Immunofluorescence Staining

Immunofluorescence staining was performed either on 5 µm thick wounded and non‐wounded skin biopsies or cultured human macrophages subjected to distinct conditions. Previously described procedures were followed.^[^ ^83^ ^]^ Primary antibodies against pSTAT1, IL‐6 (R&D system), F4/80 (e‐Bioscience), CD68, arginase‐1, pSTAT3 (Cell signaling), IGF‐1 (Millipore), K14, involucrin (Biolegend), IL‐1β (Cell signaling), TNFα (Novus) and Ki‐67 (Thermo Scientific) were used. Corresponding isotypes were used as negative controls, and AF488, AF633‐ or AF555‐conjugated goat/donkey IgG (Life Technologies) were employed as secondary antibodies. DAPI was used to counterstain nuclei. Stained tissue sections or cells were then mounted with fluorescence mounting medium (DAKO). Images were taken by a Zeiss Axiophot microscope with an AxioCam digital color camera and AxioVision software v4.8 (Carl Zeiss).

### Total RNA Isolation

RNA was isolated using RNeasy mini kit (Qiagen, Germany) from human macrophages or mouse tissues (skin or wound) subjected to the described conditions following the manufacturer's instructions. RNA concentration was measured by Qubit RNA broad range assay kit and Qubit fluorometer 3 (Thermo, USA). Prior to RNAseq analysis, RNA quality was verified by Bioanalyzer (Agilent) or Qiaxcel system (Qiagen, Germany) and RNA samples showing RIN/RIS number between 8 and 10 were subjected for further steps.^[^ ^81^ ^]^

### RNA‐Seq Library Preparation and Sequencing

High quality of RNA (1‐5 µg) from different experimental groups was subjected to rRNA depletion using Ribominus Eukaryotic system v2 kit (ThermoFisher Scientific, USA). The rRNA depleted RNA was then used for library preparation. Libraries were prepared by Illumina compatible RNA‐seq libraries using NEBNext Ultra II Directional RNA Library Preparation kit (NEB). RNA‐seq libraries were quality controlled through Qiaxcel advanced system (Qiagen) and Bioanalyzer (Agilent) and measured by Qubit fluorimeter 3 and Qubit dsDNA high sensitivity kit (ThermoFisher Scientific). Validated libraries were sequenced on Illumia NEXTSEQ 500 (1 × 75 single‐end reads) and NOVASEQ 6000 (2 × 100 paired‐end reads) systems. Data analyses were performed as described earlier.^[^ ^81^, ^84^ ^]^ In brief, demultiplexed fastq reads were aligned to the respective reference genome (mm10 for mouse and grch38 for human) using Hisat2. Aligned data (bam files) were next used for transcript assembly by cufflinks and finally cuffdiff based differential gene expression analyses were performed. The data were deposited in Gene Expression Omnibus (GEO accession: GSE183965).

### Pathway Enrichment Analysis and Hierarchical Clustering

To pull out the information from differential gene expression, hierarchical clustering analysis and visualization of analyzed data were performed as described earlier using cummRbund package and custom R scripts in Rstudio environment.^[^ ^81^, ^84^ ^]^ Gene set enrichment analysis was performed using GESA built 4.0.1. Pathway enrichment analysis was performed using Ingenuity Pathway Analysis (Qiagen, Germany).

### Phagocytosis Assay

The phagocytosis assay was performed according to the manufacturer's protocol. Briefly, macrophages from the described conditions were incubated with fluorescently labelled pHrodo Red *E. coli* (K‐12 strain, Invitrogen) or green *E. coli* (K‐12 strain, Vybrant, Invitrogen) bio‐particle for 60 min at 37 °C incubator. Then internalization of bacteria by macrophages was analyzed either through FACS or fluorescence microscopy.

### Crispr Epi‐Editing

Guide RNA was designed by CHOPCHOP (https://chopchop.cbu.uib.no/) and verified using UCSC browser. Four guide RNAs (sgRNA) per gene were selected based on score and position at the promoter region. The selected sgRNAs were synthesized through Integrated DNA technologies (IDT). 3.12 µL of each of four sgRNA (50 µm stock) (**Table** **1**) were mixed with 12.5 µL (1 µg µL^−1^) of either pcDNA‐dCas9‐p300 Core (Addgene: 61 357) or pcDNA‐dCas9‐p300 Core‐D1399Y (Addgene: 61 358). 25 µl of TransIT‐X2 was next added to the DNA/gRNA mixture. The whole mixture was mixed gently by pipette and incubated for 30 mins at room temperature. The whole mixture was then added dropwise to the macrophages on a 10 cm^2^ cell culture plate. After 48 h, the transfected cells were washed and treated with sodium palmitate and sodium butyrate for 12 h, followed by total RNA isolation from these cells using RNeasy mini kit (Qiagen). 1 µg of total RNA was transcribed to cDNA using Ready‐to‐go RT‐PCR beads (Cytiva). The cDNA was used to study expression of IRF1 and S100A8 (**Table** **2**) using Syber Green based detection in a QuantStudio 5 system (Thermo).

**Table 1:** List of gRNA sequence.

	gRNA sequence
IRF1_sgRNA1	GTGGACCGCCCACCCGGACG
IRF1_ sgRNA2	CGCTAAGTGTTTGGATTGCT
IRF1_ sgRNA3	TTGTAGAGCTAGCGGCGAAG
IRF1_ sgRNA4	TCCTCCGGGCAAGCCGGAGC
S100A8_sgRNA1	GGATGATAGGGATACACGTG
S100A8_ sgRNA2	ATGTGCCCTTACCCACTGGA
S100A8_ sgRNA3	CAAGCCTAACCGCTATAAAA
S100A8_ sgRNA4	TAGCAATCGCAATAGTGGAG

**Table 2:** List of primers.

	qPCR primers
IRF1_FP	GGGCTCATCTGGATTAATAAAGAGG
IRF1_RP	CTTGTTGATGTCCCAGCCATG
	
S100A8_FP	TGAAGAAATTGCTAGAGACCGAGTG
S100A8_RP	ATCCAACTCTTTGAACCAGACGTC
	
ACTB_FP	CCTTCCTTCCTGGGCATGG
ACTB_RP	GTTGGCGTACAGGTCTTTGC

### Skin and Wound Tissue Digestion to Prepare Single Cell Suspension

Unwounded and wounded skins from mice were collected and washed in ice‐cold PBS. These freshly harvested tissues were then finely minced and subjected to enzymatic digestion by incubating with 5 mL of digestion solution (0.350 mg mL^−1^ liberase TL, 0.1 mg mL^−1^ DNase in serum‐free RPMI) for 30 min at 37 °C while rocking at 350 rpm. After 30 min, 0.5 ml of 0.25% Trypsin‐1 mM EDTA was added into digestion reaction and incubated further for 10 min. Digestion was stopped with 10 mL RPMI, supplemented with 10% FCS, samples were vortexed for a few seconds, and then strained through 70 and 40 µm sieves and centrifuged to collect the cells. The cells were resuspended in ice‐cold PBS, followed by the addition of debris removal solution (Miltenyi biotec) and overlay with ice‐cold PBS. Following centrifugation, the precipitated cells without cell and matrix debris, were resuspended in 100 µL of dead cell removal microbeads (Miltenyi biotec) and passed through MS column to collect the live cells, which were either used for flow cytometry and

### Flow Cytometry

Single‐cell suspensions were washed in FACS staining buffer (2% FCS in PBS with 0.48 mm EDTA) and suspended in staining buffer (100 µL per 10^6^ cells) containing TruStain FcX to block FC receptors and Monocyte blocker to block non‐specific binding of tandem dyes to monocyte/macrophage. Then desired fluorophore‐conjugated antibody cocktails were added to the blocked cells and incubated for 30 min for staining the surface markers. After staining the surface markers, the cells were washed with staining buffer and fixed and permeabilized using Foxp3 Fixation/Permeabilization kit (Thermo), followed by the addition of antibody cocktails for staining intracellular markers and stained for 30 min. The details of the antibodies were presented in the **Table** **3**. Cells were also stained with respective isotype control fluorophore conjugated antibodies. After final staining, cells were washed with staining buffer and finally resuspended in 300 µL staining buffer. Cells were also stained with each fluorochrome individually to use in the compensation setting. All samples were run on a BD LSRII cytometer (BD Biosciences) equipped with violet, blue, and red lasers and analyzed in the BD FACS Diva analyzer suite (BD Biosciences). For all cell types, the initial gate from forward scatter (FSC‐A) versus side‐scatter (SSC‐A) plots was used. From this, single‐cell gate from FSC‐A versus FSC‐H plots was used to exclude debris. Strict doublet exclusion was performed prior gating for immune cells (CD45^+^). Cytotoxicity of SP or butyrate was determined by measuring the intensity of the fluorescent Calcein dye (Invitrogen) taken up by viable macrophages. Analysis and visualization of flow cytometry data were performed using FlowJo (Version 9) and FlowLogic (Version 1.0).

**Table 3:** List of antibodies used for FACS analysis.

Antibody	Company	Catalogue No
Alexa Fluor 488 anti‐mouse CD36	Biolegend	102 607
Alexa Fluor 488 Armenian Hamster IgG Isotype	Biolegend	400 923
PE/Dazzle 594 anti‐mouse CD163 Antibody	Biolegend	155 315
PE/Dazzle 594 Rat IgG2a, κ Isotype Ctrl Antibody	Biolegend	400 557
PE/Cyanine7 anti‐mouse CD206 (MMR)	Biolegend	141 719
PE/Cyanine7 Rat IgG2a, κ Isotype	Biolegend	400 521
APC anti‐mouse/human CD11b	Biolegend	101 212
APC Rat IgG2b, κ Isotype	Biolegend	400 611
PE/Cyanine5 anti‐mouse CD86	Biolegend	105 015
PE/Cyanine5 Rat IgG2a, κ Isotype	Biolegend	400 509
Brilliant Violet 605 anti‐mouse CD68	Biolegend	137 021
Brilliant Violet 605 Rat IgG2a, κ Isotype	Biolegend	400 539
PE anti‐mouse Arginase 1	Biolegend	165 803
PE Rat IgG2b, κ Isotype	Biolegend	400 635
Brilliant Violet 421 anti‐mouse CD45	Biolegend	103 133
Brilliant Violet 421 Rat IgG2b, κ Isotype	Biolegend	400 639
PE/Cyanine7 anti‐mouse F4/80	Biolegend	123 113
PE/Cyanine7 Rat IgG2a, κ Isotype	Biolegend	400 521

### Single Cell RNA Sequencing and Bioinformatic Analysis

Single cell RNA sequencing was performed using Evercode WT v3 single cell whole transcriptome kit (Parse biosciences, USA), as per manufacturer instructions. In brief, single cell suspensions (48 000 cells from each sample) from unwounded (Day 0) and post‐wound (Day 5 and Day 10) mouse skin, were first fixed and permeabilized followed by cell barcoding, cDNA synthesis, and library preparation. Single cells from each group were suspended in cell prefixation master mix, followed by addition of cell fixative and incubation at ice for 10 min to fix the cells. The fixed cells were then permeabilized by adding permeabilization solution. After washing the cells with fix and permeabilization stop buffer, cells were resuspended in cell storage master mix, containing 5% DMSO, and then passed through 40 µm cell strainer and either processed directly for cell barcoding or stored at −80 °C.

After counting, fixed and permeabilized cells were in situ barcoded using four rounds of split‐pool barcoding techniques. In the first barcoding reaction, cells were subjected to round 1 cell barcode mediated first strand cDNA synthesis (reverse transcription). After 1st barcoded reverse transcription (first strand of cDNA synthesis), cells were pooled and distributed into each well of a 96 well plate, containing unique 2nd round of barcode in each of the well. After the second round of barcoding, cells were again pooled and distributed into another 96 well plate for 3rd round of barcoding. The addition of reagents to the plates was performed by automatic noncontact dispenser I.DOT (Dispendix). Following 3rd round of barcoding, the cells were again pooled and distributed equally into 8 strip PCR tubes (sub‐libraries) to lyse and capture biotinylated cDNA using streptavidin beads. cDNA template switching was next performed to add an adapter to the 3′ end of the captured cDNA. cDNA amplification was next performed using template switch oligo specific primer and 5′ of 3rd round of barcoding oligo specific primer. Purified amplified double stranded cDNA was next fragmented (≈250 bp) and end repaired, followed by size selection using SPRI beads. The size selected fragmented, end‐repaired cDNA was then used to add 5′ adapter with Illumina Truseq R2 sequence, followed by round 4 barcoding reaction. Post barcoding, the library was purified, size selected, and quantified using Qubit dsDNA HS assay kit (Thermo). The library was next subjected to quality control and to check size distribution using QIAxcel Advanced capillary electrophoresis system using high resolution kit.

The libraries were then sequenced in NovaSeq 6000 using S2 reagent kit with read 1 for 64 cycles, i7 index 1 for 8 cycles, i5 index 2 for 8 cycles, and read 2 for 58 cycles. Read 1 contains the transcript sequence, while read 2 contains the 10 bp polyN sequence and the three 8 bp plate barcodes separated by two linker sequences. The demultiplex raw reads (fastq) were next used for genome alignment and transcript discovery, quantification and per identified barcode reporting. The fastq files from each sub‐library were first aligned to mouse genome (GRCm39) using customized scripts (split‐pipe, version 1.3.1), which uses STAR aligner (2.7.11b) to align the fastq reads. The gene expression matrix (count matrix), and barcode list (cell_metadata), and gene information (all_genes) were finally generated by split‐pipe scripts. Around 48 000 quality filtered total cells were taken for analyses. The count matrix, cell_metadata (cell barcode information) and all_genes (gene annotation) were next loaded into RStudio environment (2004.04.2, build 764) for the data analyses using Seurat (version 5.1.0). Cell‐cell interactions between the cell clusters were analyzed by CellChat (version 2.1.2). The visualization of the data analyses was performed by ggplot2, plot1cell, dittoseq packages. Pseudobulk differential expression analyses between different groups were analyzed by DESeq2. Gene set enrichment analyses were performed by fgsea and escape packages. The data analyses were performed using HPC clusters, bwUniCluster 2.0 and bwForCluster BinAC from Baden‐Württemberg, Germany (bwHPC).

### Statistical Calculations

Error bars represent SEM. The significance of differences between two groups was analyzed by Student's *t* test or one‐way ANOVA for comparing the difference between more than two groups and presented as *P*‐values.

## Conflict of Interest

The authors declare no conflict of interest.

## Author Contributions

P.M. and K.S‐K., contributed equally to this work. K.S. conceived and designed the study, performed experiments, and drafted the manuscript. A.K.K. R.K.P., Y.W., J.C. P.H. M.A. and M.W. contributed in designing and technically supporting the experiments. L.K. and A.H. technically supported this work. P.M. designed the experiments and was involved in bioinformatic data analysis. K.S.K. supervised the study and wrote the manuscript. All authors read and approved the final manuscript.

## Supporting information



Supporting Information

## Data Availability

The RNA‐Seq data used in this present study were deposited in the Gene Expression Omnibus (GEO) with accession number “GSE183965”. Publicly available data sets “GSE165816” and “GSE154345” were used to analyze human diabetic wounds and macrophage polarization.
