# Supplementary material for: Restoring Histone Acetylation Accelerates Diabetic Wound Repair by Improving the Spatiotemporal Dynamics of Macrophages
Source: Adv Sci (Weinh). 2025 Oct 6;12(46):e04920. doi: 10.1002/advs.202504920 (PMC12697871; doi:10.1002/advs.202504920)
Supplement: Supplementary file 1 — Supporting Information [file ADVS-12-e04920-s001.docx]

**Supplementary information**
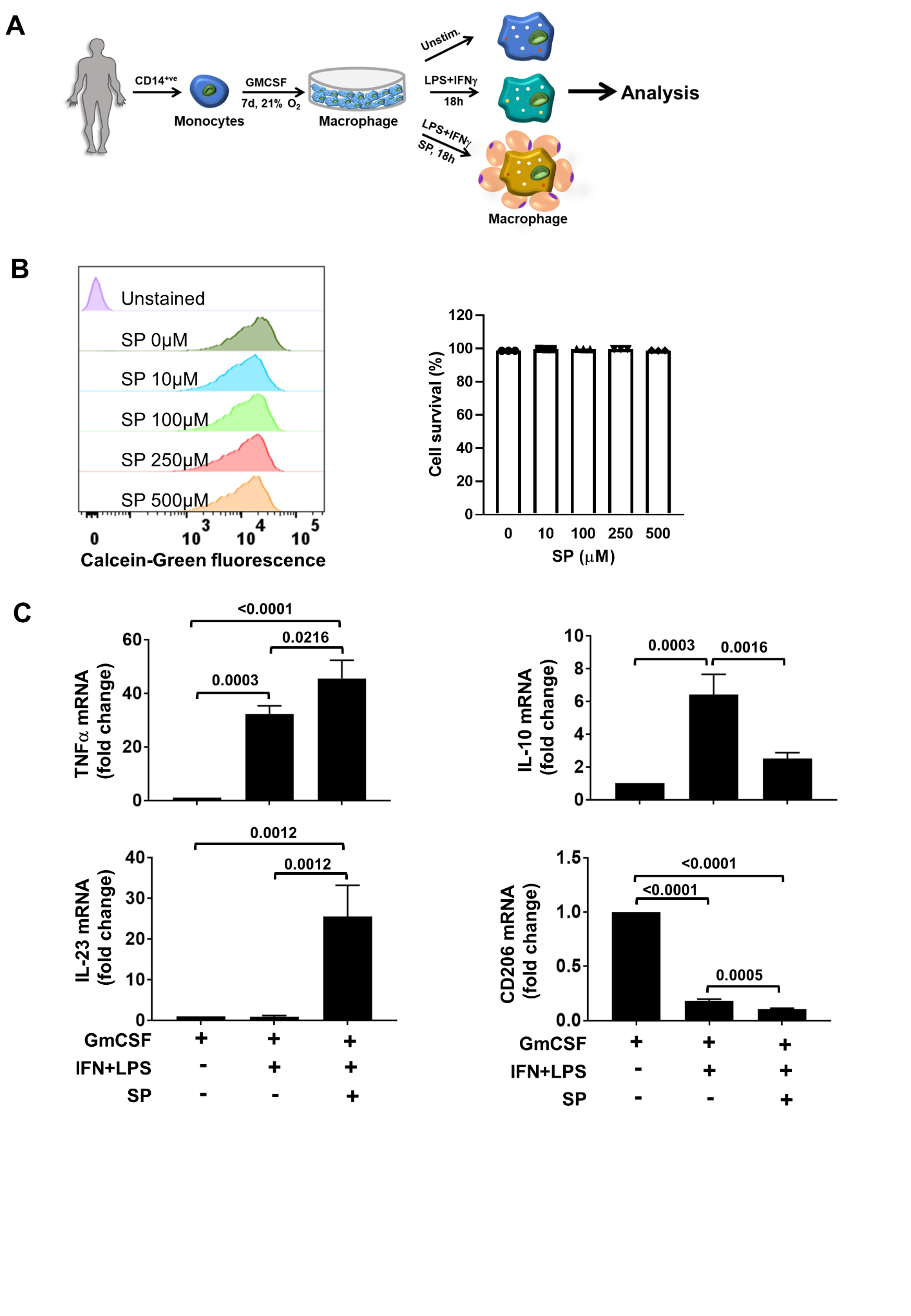


**Supplementary Figure 1. Sodium palmitate abrogates LPS and IFNγ associated signaling in human macrophages.**

**(A)** Schematic representation of experimental setup describing different conditions of studied human macrophage responses. **(B)** Calcein-Green fluorescence depicting cell viability following incubation with different SP concentrations. One-way ANOVA, values are represented as mean ± SEM, n=3.   **(C)** Quantitative RT-PCR depicting expression of different genes linked to macrophage polarization following 12h incubation with the saturated free fatty acid SP. Macrophage differentiation from human buffy coat monocytes (CD14^+ve^) was induced either with GmCSF after subjecting them to culture for 7 days. These differentiated macrophages were then stimulated with LPS (10 ng/ml) and IFNγ (20 ng/ml) in the presence or absence of 250µM SP. One-way ANOVA, values are represented as mean ± SEM, n=3.


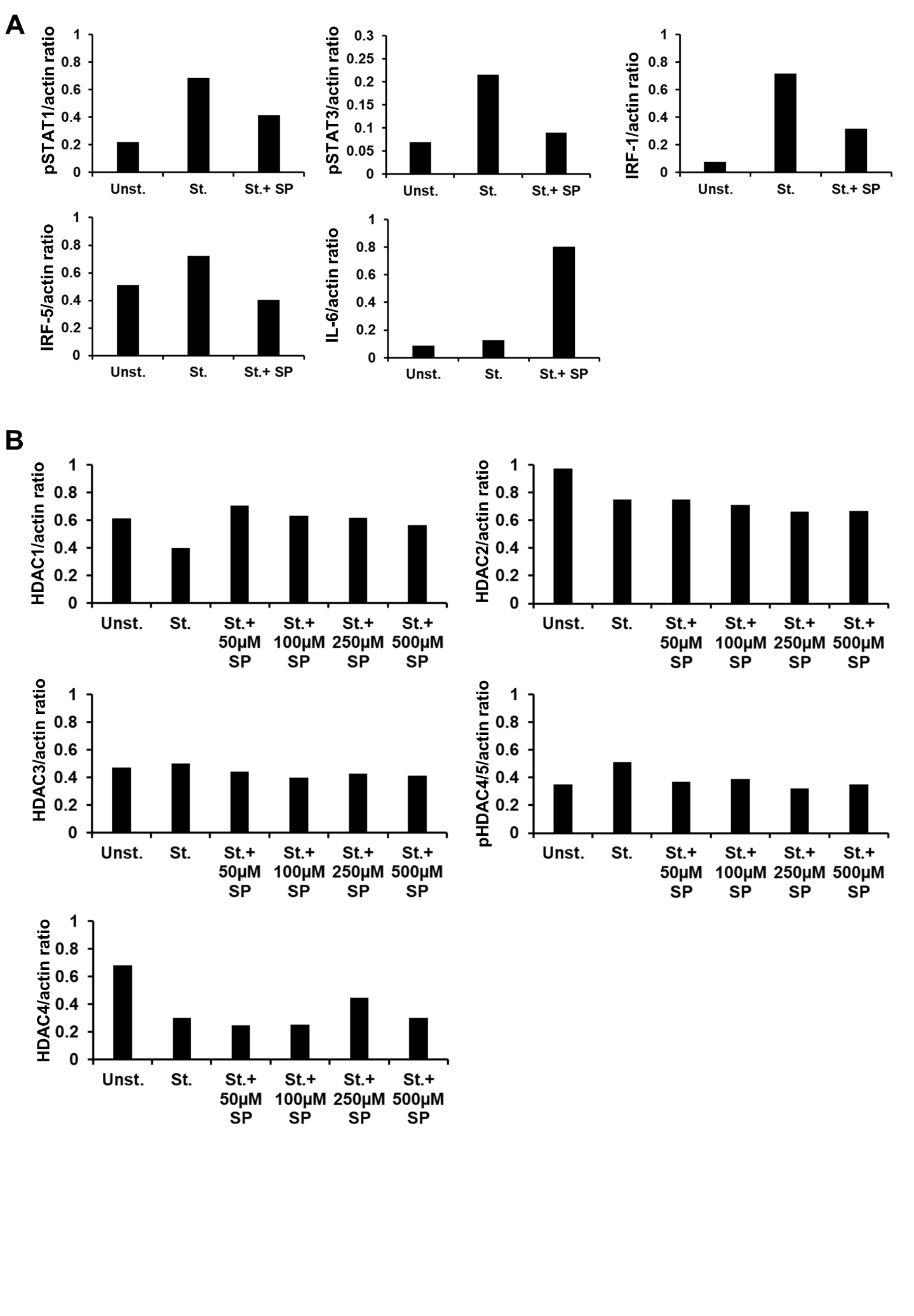


**Supplementary Figure 2. Sodium palmitate suppresses STAT1 signaling and modulates the expression of HDAC family members.**

**(A)** Densitometry analysis of western blot images depicted in Figure 1D. **(B)** Densitometry analysis of western blot images depicted in Figure 1G.


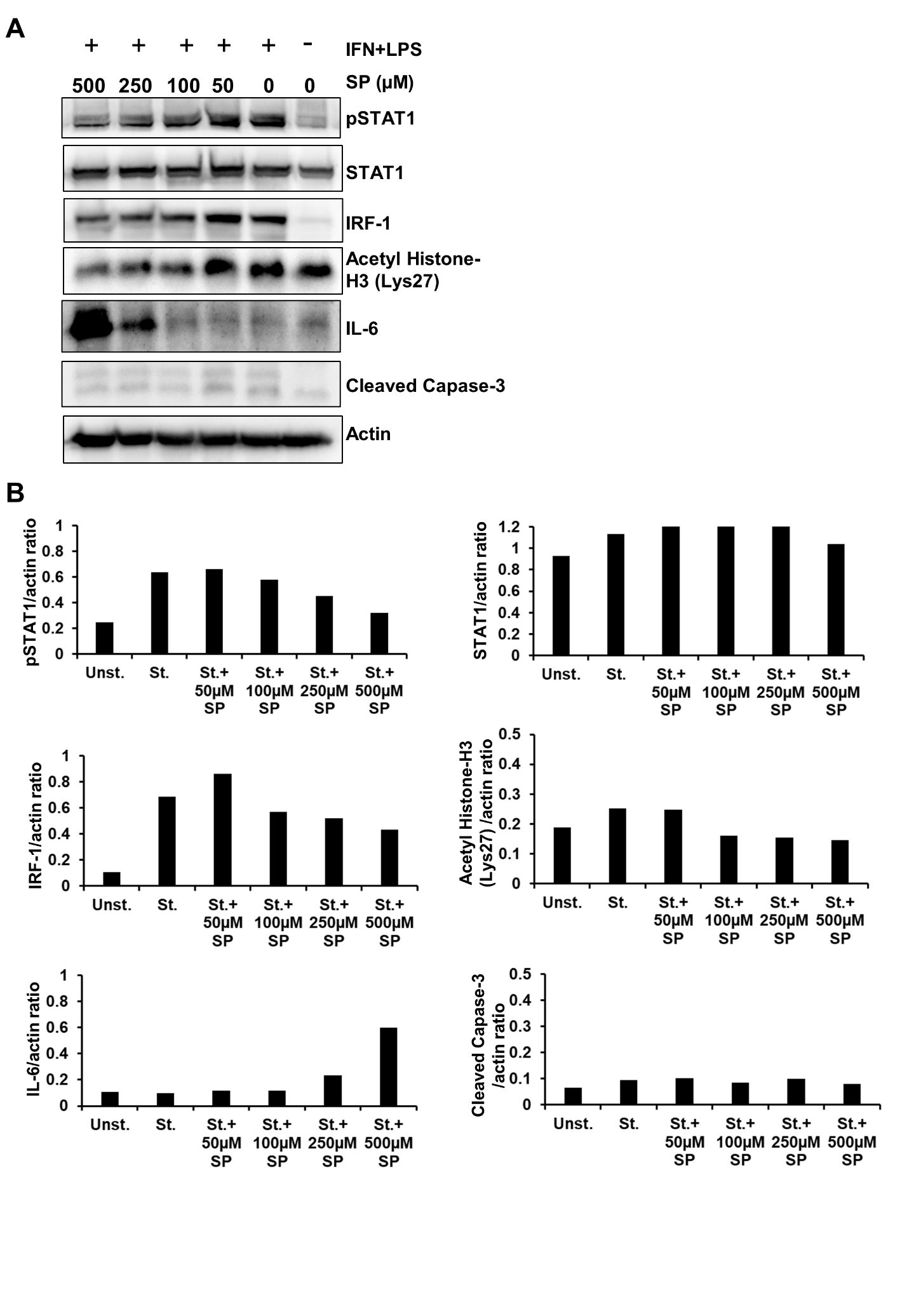


**Supplementary Figure 3. Sodium palmitate inhibits acetylation of histone core in the macrophages.**

**(A)** Western blot analysis displaying expression of key proteins regulating inflammatory signaling after incubation of stimulated macrophages with increasing concentrations of sodium palmitate for 18h. These results are representative of three independent experiments. Human beta-actin was used as a loading control. **(B)** Densitometry analysis of western blot images depicted in Supplementary Figure 3A.


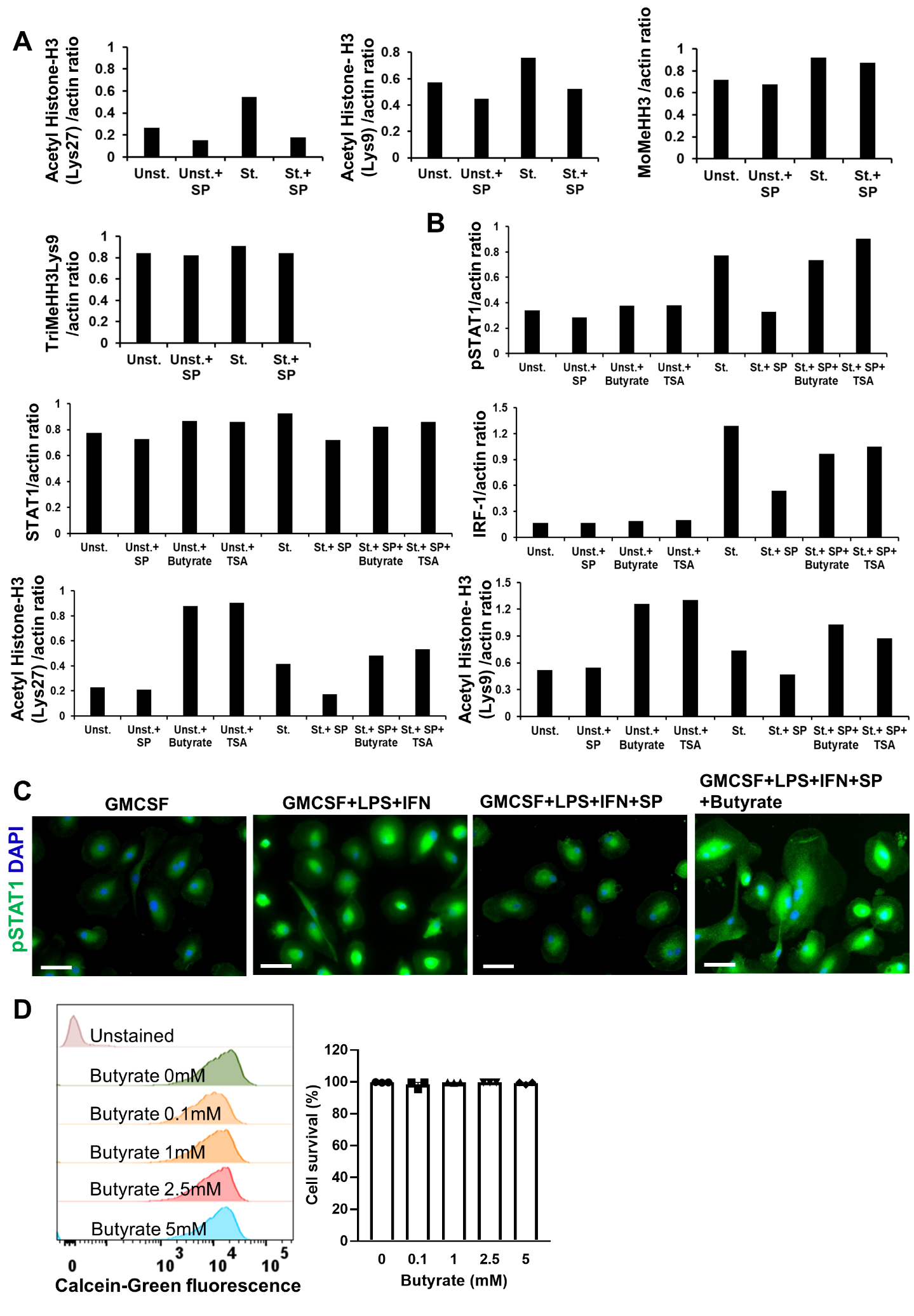


**Supplementary Figure 4. Butyrate attenuates sodium palmitate mediated impairment of macrophage signaling.**

**(A)** Densitometry analysis of western blot images depicted in Figure 2A. **(B)** Densitometry analysis of western blot images depicted in Figure 2B. **(C)** Immunostaining depicting differential expression of pSTAT1 (green) in cytoplasm and nucleus of macrophages exposed to the indicated conditions, nuclei were counterstained with DAPI. Scale bars, 50µm (n=3). SP, Sodium palmitate. **(D)** Calcein-Green fluorescence depicting cell viability following incubation with different butyrate concentrations. One-way ANOVA, values are represented as mean ± SEM, n=3.


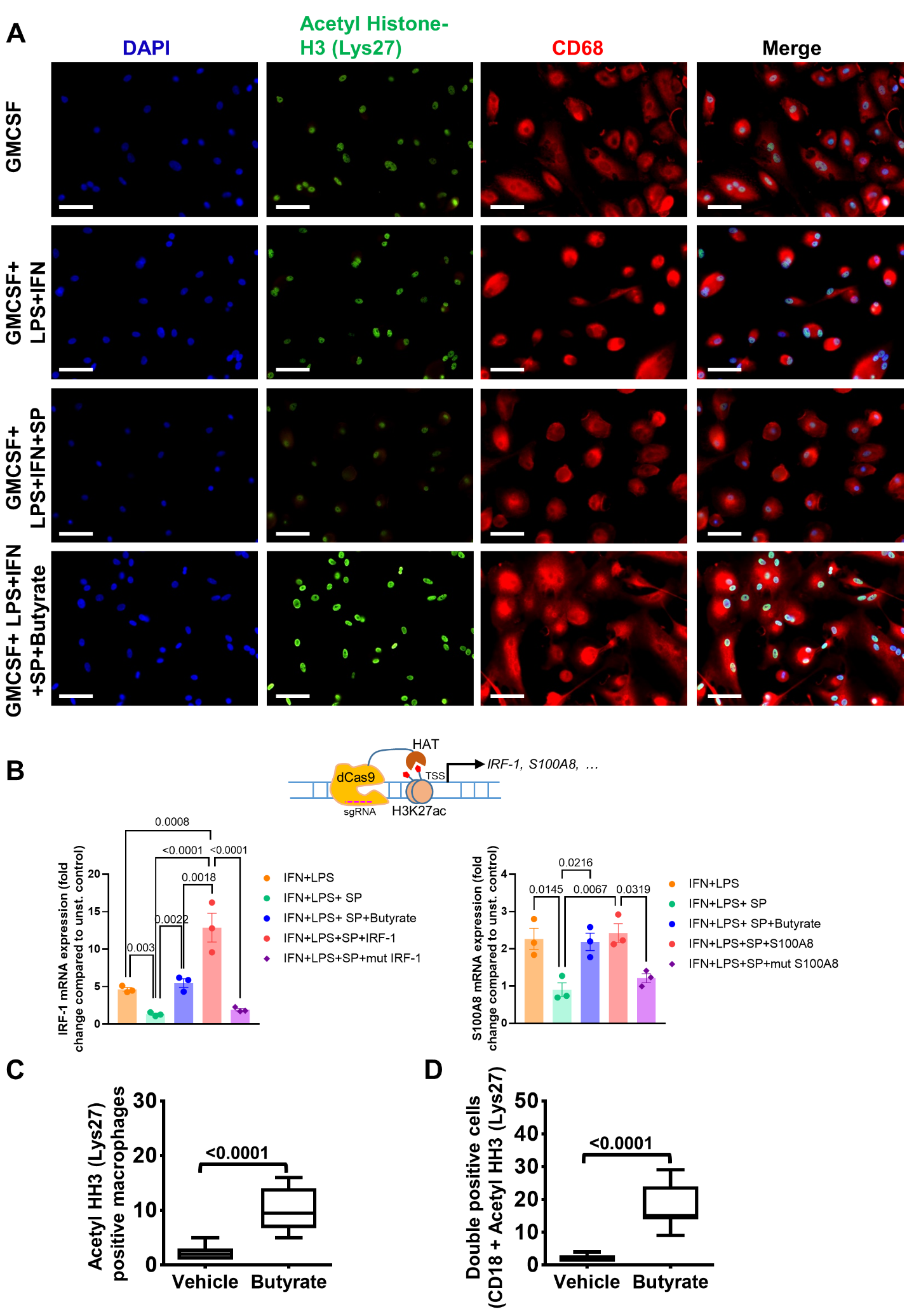


**Supplementary Figure 5. Acetylation of histone core is essential for enhanced macrophages activity under diabetic conditions.**

**(A)** Immunostaining depicting differential expression of acetyl histone-H3 (Lys27) (green) in the nucleus of macrophages (CD68, red) exposed to the indicated conditions, nuclei were counterstained with DAPI (blue). Scale bars, 200µm (n=3). CD68 was used to mark macrophages (red). Nuclei were counterstained with DAPI (blue). Scale bars, 50µm (n=3). SP, Sodium palmitate. **(B)** Direct induction of acetyl histone-H3 (Lys27) at IRF-1 and S100A8 locus through dCas9-HAT in SP exposed macrophages. One-way ANOVA, values are represented as mean ± SEM, n=3. **(C-D)** Quantitative analysis of immunostaining images depicted in Figure 2E and 2F. Unpaired t-test, values are represented as mean ± SEM, n=5.


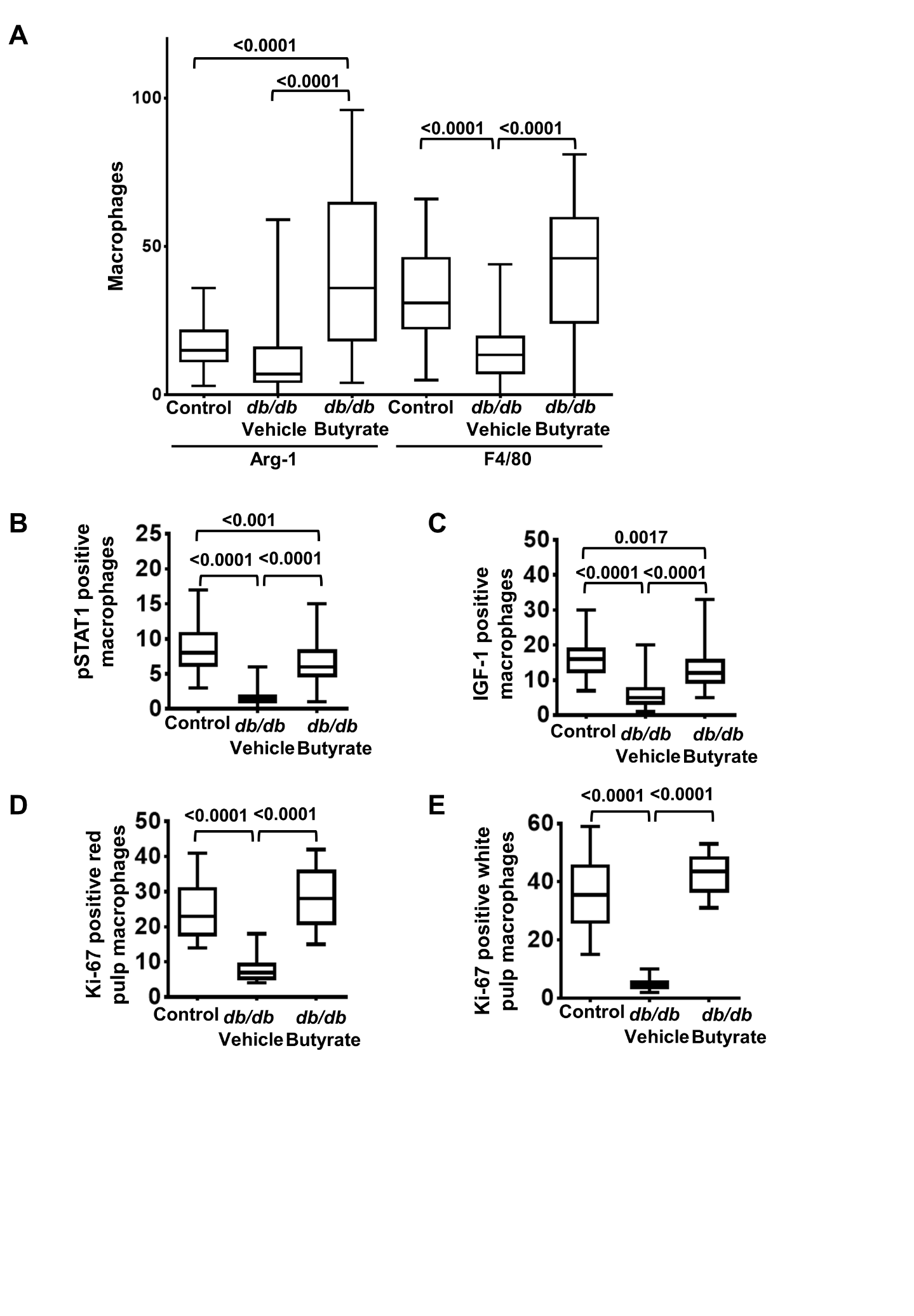


**Supplementary Figure 6. HDAC inhibitor butyrate promote macrophage recruitment and activity in diabetic mice.**

**(A)** Quantitative analysis of Figure 4A, quantification is performed in the images captured with 40X objective. One-way ANOVA, values are represented as mean ± SEM, n=5. **(B-E)** Quantitative analysis of immunostaining images depicted in Figure 4B-C and 4F. One-way ANOVA, values are represented as mean ± SEM, n=5.


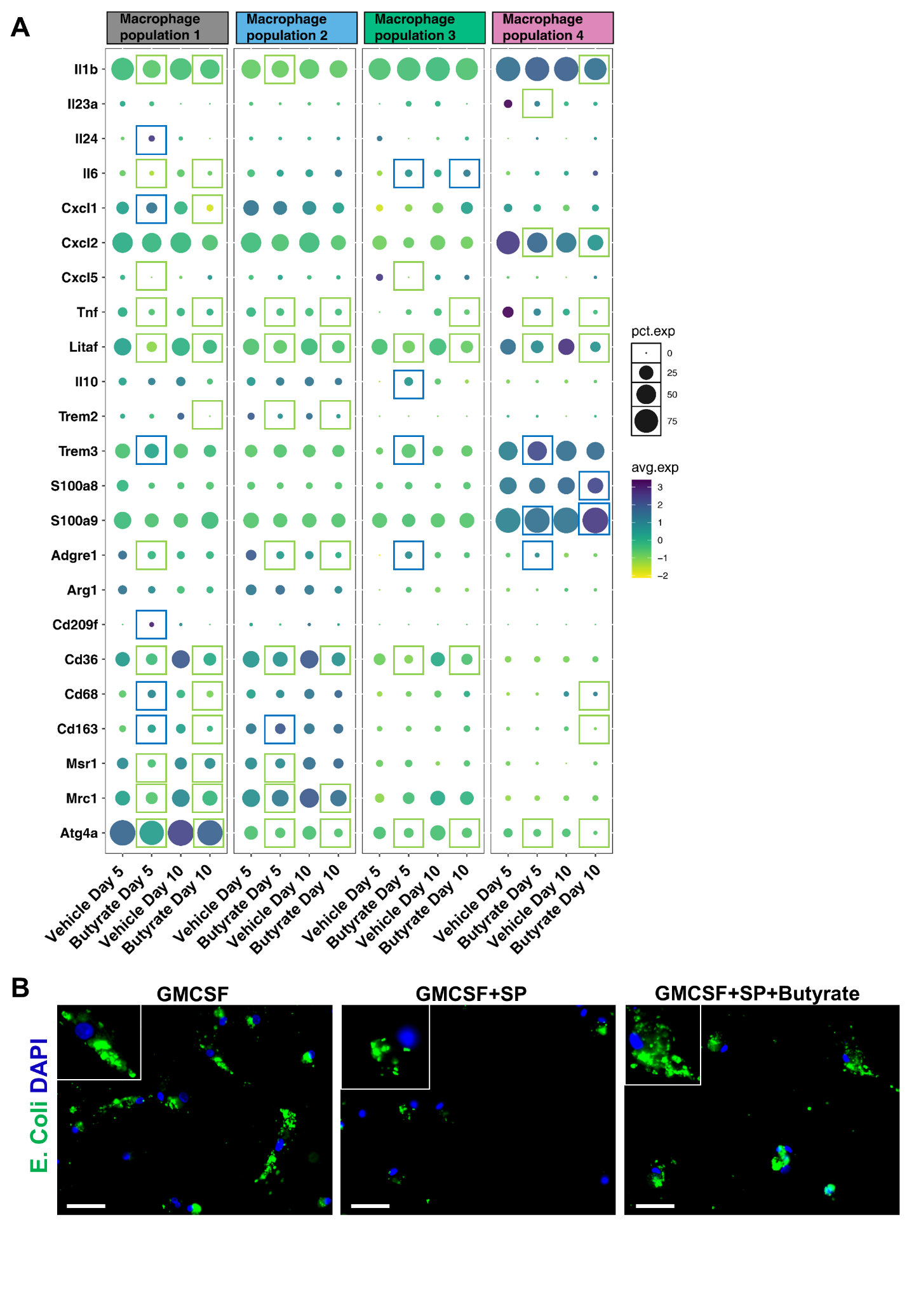


**Supplementary Figure 7. Butyrate rescues deviant inflammatory signaling and impaired macrophage phagocytosis in wounds of diabetic mice.**

**(A)** Dot plot showing variance-scaled, percent of cells expressing (dot size) pro- and anti-inflammatory genes and mean expression (dot color) in four different macrophages subpopulation of *db/db* mice wounds. Red square labels indicate upregulated and green square labels indicate downregulated genes by butyrate versus vehicle*.* **(B)** Phagocytosis assay displaying the uptake of pHrodo Green *E. coli* after incubation with human macrophages from distinct treatment groups for 60 min at 37°C. The pHrodo dyes are essentially non-fluorescent at neutral pH and exhibit increasing green fluorescent signal as the pH decreases. Scale bars, 20µm (n=3).


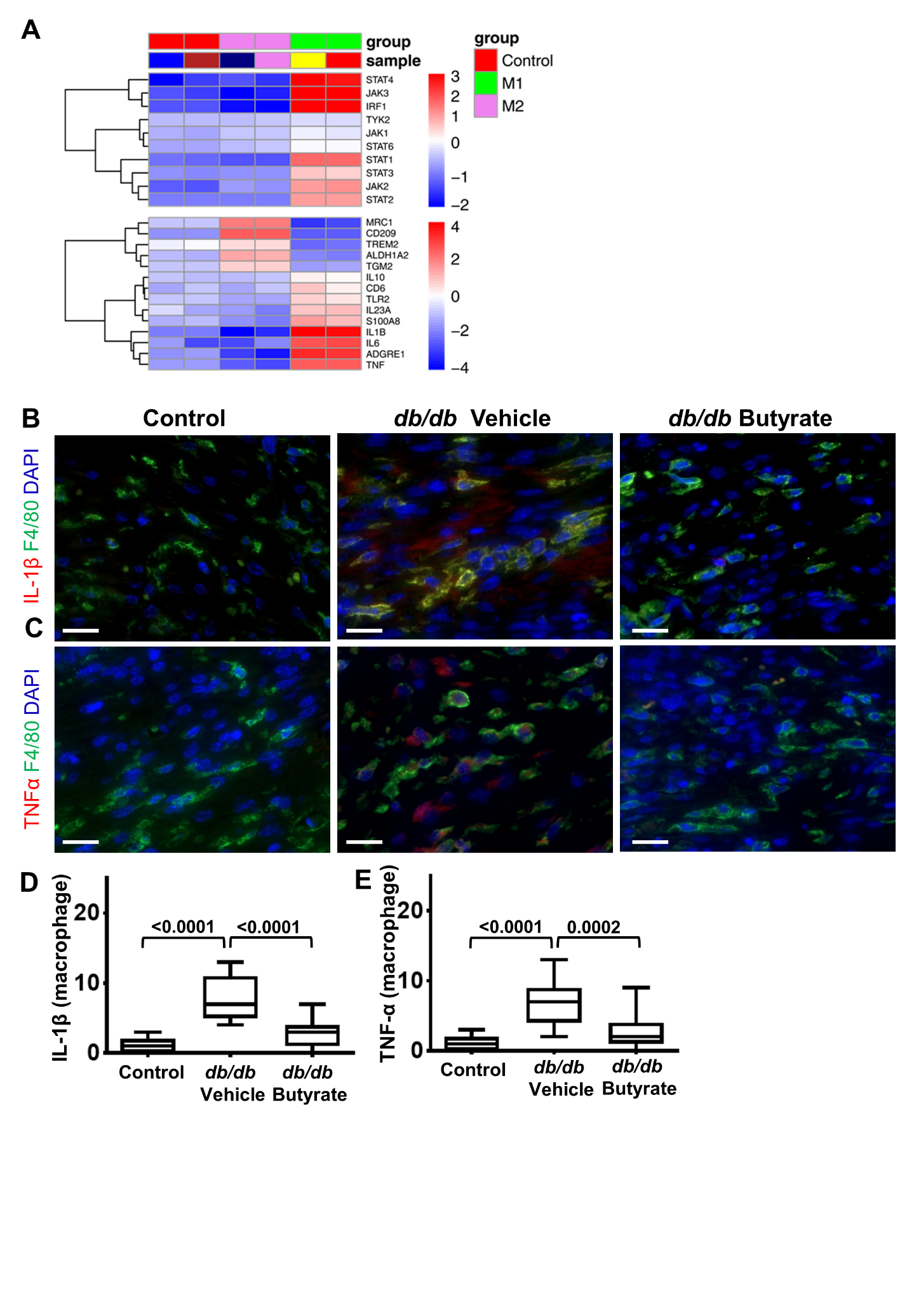


**Supplementary Figure 8. Butyrate mediated restoration of histone acetylation in macrophages restricts chronic inflammation in diabetic wounds.**

**(A)** Gene expression analysis of publicly available data sets from macrophages derived from the human monocytic cell line THP-1. These macrophages were subjected to phorbol myristate acetate for 24 h to generate M0 macrophages (control) or to IL-4 to generate M2 macrophages or to IFNγ/LPS to generate M1 macrophages. Color code indicates relative expression. **(B)** Representative immunostaining microphotographs of IL-1β (red), **(C)** TNFα (red) and pan macrophage marker F4/80 (green) in 10 days old wound sections of either control, butyrate or vehicle treated *db/db* mice and **(D-E)** their quantification. Cell nuclei are stained with DAPI (blue). Scale bar, 20µM. One-way ANOVA, values are represented as mean ± SEM, n=5.


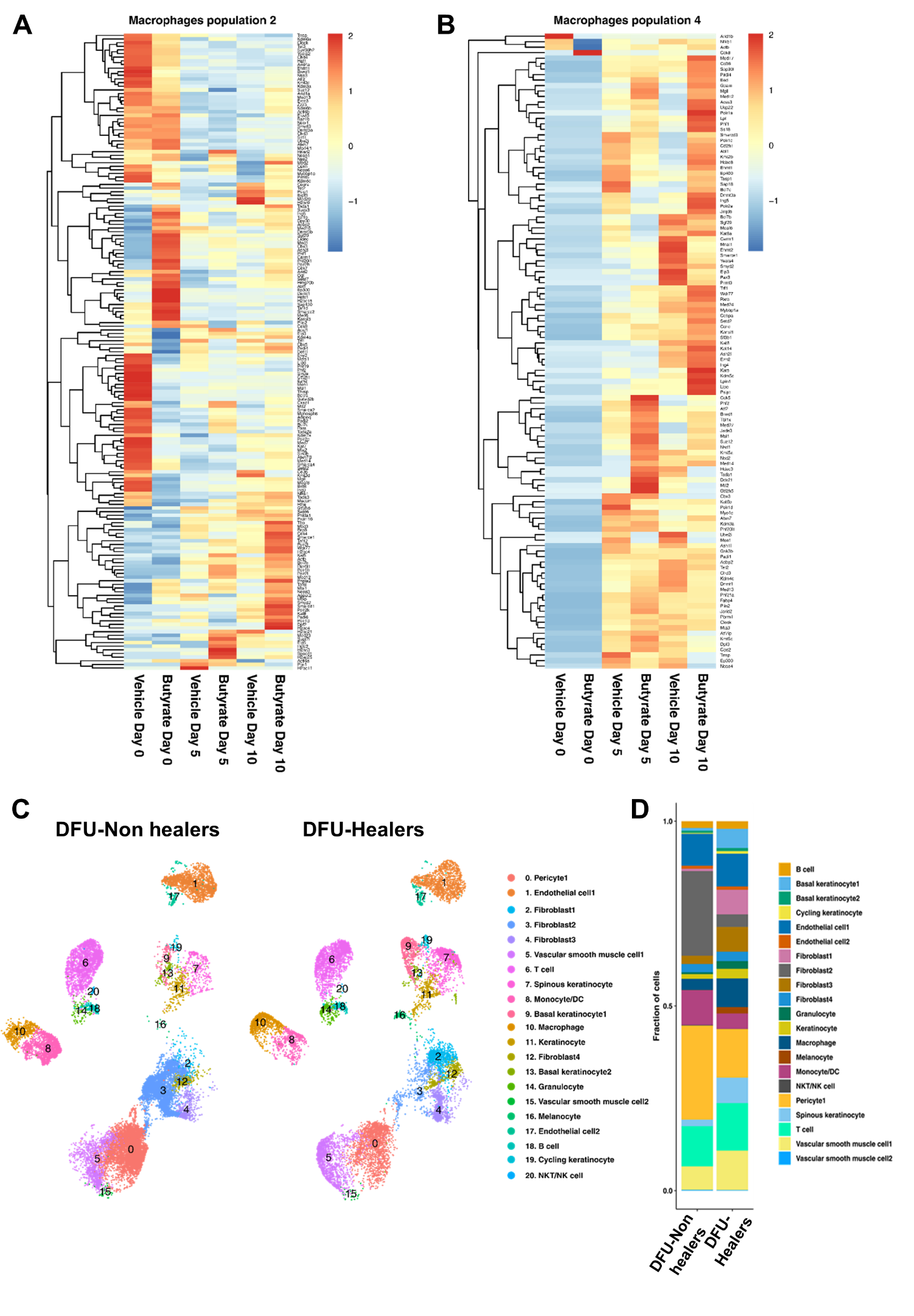


**Supplementary Figure 9. Epigenetic profiles of macrophages and repair pathways changes in wounds of diabetic mice treated with butyrate.**

**(A)** Reactome/GSEA analysis algorithms demonstrating the epigenetic transcriptome of ‘macrophages population 2’ and **(B)** ‘macrophages population 4’ obtained from diabetic wounds treated either with butyrate or vehicle. Color code indicates relative expression.

**(C-D)** UMAP and stacked bar plots derived from publicly available single cell RNAseq data to visualize differences between DFU-Healers and DFU-Non healers cells.


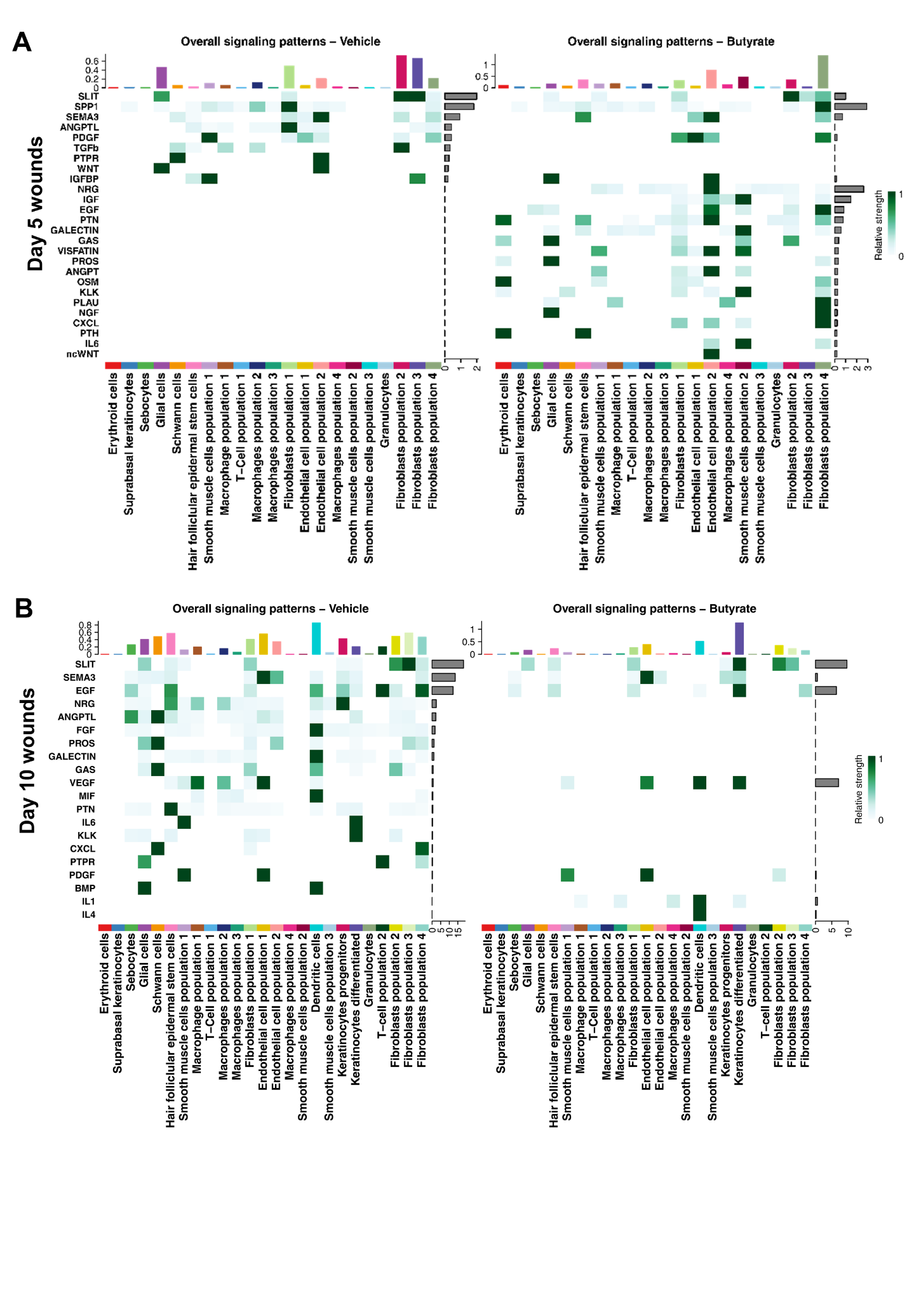


**Supplementary Figure 10. Butyrate remodels the signaling pattern of wound resident cells in diabetic mice.**

**(A)** Heatmap derived from CellChat showing the kinetics of butyrate activated secreted signaling pathways in diverse cell type from D5 wounds versus vehicle. Color code indicates relative number. **(B)** Heatmap derived from CellChat showing the kinetics of butyrate activated secreted signaling pathways in diverse cell type from D10 wounds versus vehicle. Color code indicates relative number.


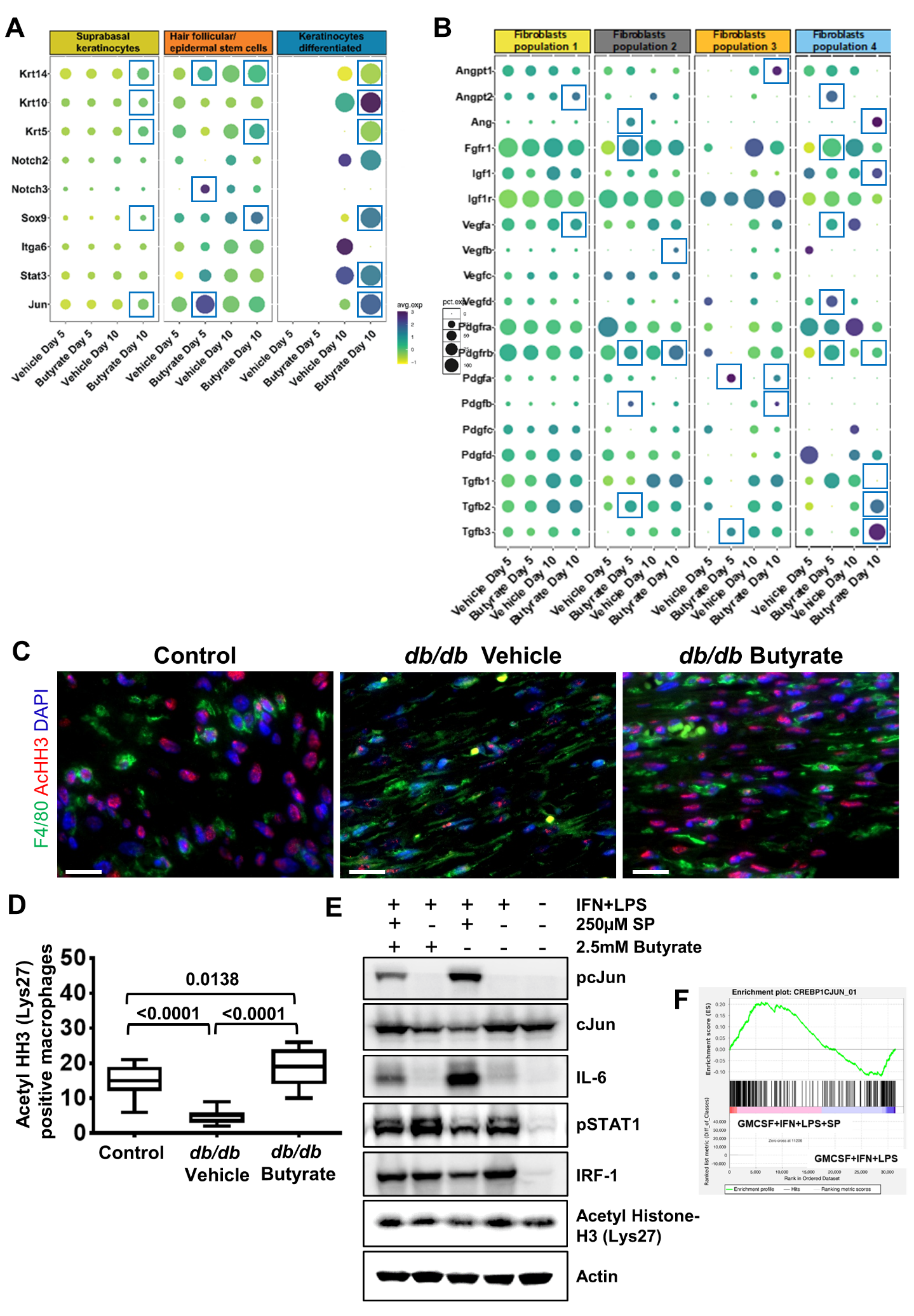


**Supplementary Figure 11. Restoration of histone acetylation promotes tissue repair by harmonizing communication among wound resident cells.**

**(A)** Dot plot showing variance-scaled, percent of cells expressing (dot size) genes and mean expression (dot color) in three keratinocyte subsets and **(B)** four different fibroblasts subpopulation of *db/db* mice wounds. Red square labels indicate upregulated genes by butyrate. **(C-D)** Representative immunostaining microphotographs of acetyl histone-H3 (Lys27) (red) and pan macrophage marker F4/80 (green) in 10 days old wound sections of either control, butyrate or vehicle treated *db/db* mice. Scale bar, 20µM. One-way ANOVA, values are represented as mean ± SEM, n=5. **(E)** Butyrate dampens SP-induced upregulation of AP1 transcription factor pcJUN, while restoring activated STAT1 signaling to increased levels of stimulated macrophages in the presence of SP. These results are representative of three independent experiments. Human beta-actin was used as a loading control. **(F)** cJUN family gene in stimulated macrophages following exposure with SP.
